# A Photoresponsive Homing Endonuclease for Programmed DNA Cleavage

**DOI:** 10.1021/acssynbio.3c00425

**Published:** 2023-12-07

**Authors:** Luke A. Johnson, Robert J. Mart, Rudolf K. Allemann

**Affiliations:** School of Chemistry, Cardiff University, Main Building, Park Place, CF10 3AT, Cardiff, U.K.

**Keywords:** homing endonuclease, light-oxygen-voltage domain, DNA cleavage, light-induced dimerization, programmable nuclease, photoresponsive

## Abstract

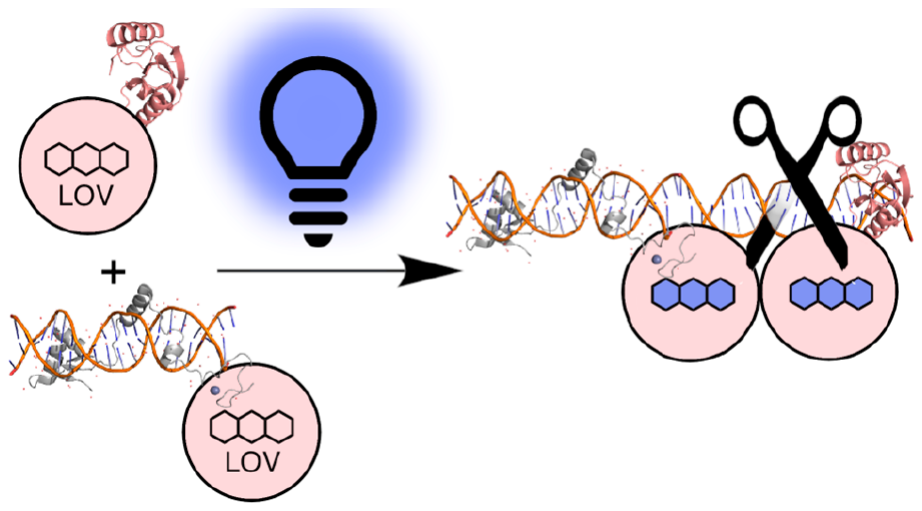

Homing endonucleases are used in a wide range of biotechnological applications including gene editing, in gene drive systems, and for the modification of DNA structures, arrays, and prodrugs. However, controlling nuclease activity and sequence specificity remain key challenges when developing new tools. Here a photoresponsive homing endonuclease was engineered for optical control of DNA cleavage by partitioning DNA binding and nuclease domains of the monomeric homing endonuclease I-TevI into independent polypeptide chains. Use of the Aureochrome1a light-oxygen-voltage domain delivered control of dimerization with light. Illumination reduced the concentration needed to achieve 50% cleavage of the homing target site by 6-fold when compared to the dark state, resulting in an up to 9-fold difference in final yields between cleavage products. I-TevI nucleases with and without a native I-TevI zinc finger motif displayed different nuclease activity and sequence preference impacting the promiscuity of the nuclease domain. By harnessing an alternative DNA binding domain, target preference was reprogrammed only when the nuclease lacked the I-TevI zinc finger motif. This work establishes a first-generation photoresponsive platform for spatiotemporal activation of DNA cleavage.

## Introduction

Homing endonucleases promote the site-directed integration of mobile genetic elements by single- or double-strand DNA breaks.^1,2^ To ensure the integrity of the host’s genome, homing endonucleases are typically encoded within self-splicing introns or inteins and have lengthy recognition sites (14–44 bp).^3^ The resulting high specificity makes homing endonucleases particularly suited for genome editing and gene drive applications.^4−7^ Tailoring the sequence specificity of homing endonucleases, however, remains a significant challenge for protein engineering. Where other classes of nucleases such as engineered zinc-finger nucleases (ZFNs)^8,9^ and transcription activator-like effector nucleases (TALENs)^10−12^ as well as clustered regularly interspaced short palindromic repeats (CRISPR)^13−15^ associated nucleases can be targeted using predictable protein:DNA and RNA:DNA interactions, homing endonucleases typically have less predictable interactions with DNA and often require in vivo selection methods to modify target specificity.^7,16^ Hybrid homing endonucleases have therefore been developed that leverage this predictability by combining elements of ZFNs, TALENs, and the CRISPR-associated enzyme 9 (Cas9).^17−20^ The success of these programmable endonucleases has led to applications in diverse fields outside of genome editing and gene drive systems including in biomaterials,^21^ biological sensing,^22^ clinical diagnostics,^23^ and biosecurity.^24^ Despite the widespread use of CRISPR-based tools, TALENs and homing endonucleases outperform CRISPR nuclease systems in certain uses due to their differences in activity and the mechanism of locating target sequences.^25,26^ In all such applications controlling nuclease activity and sequence specificity is fundamental.

**Figure 1:**
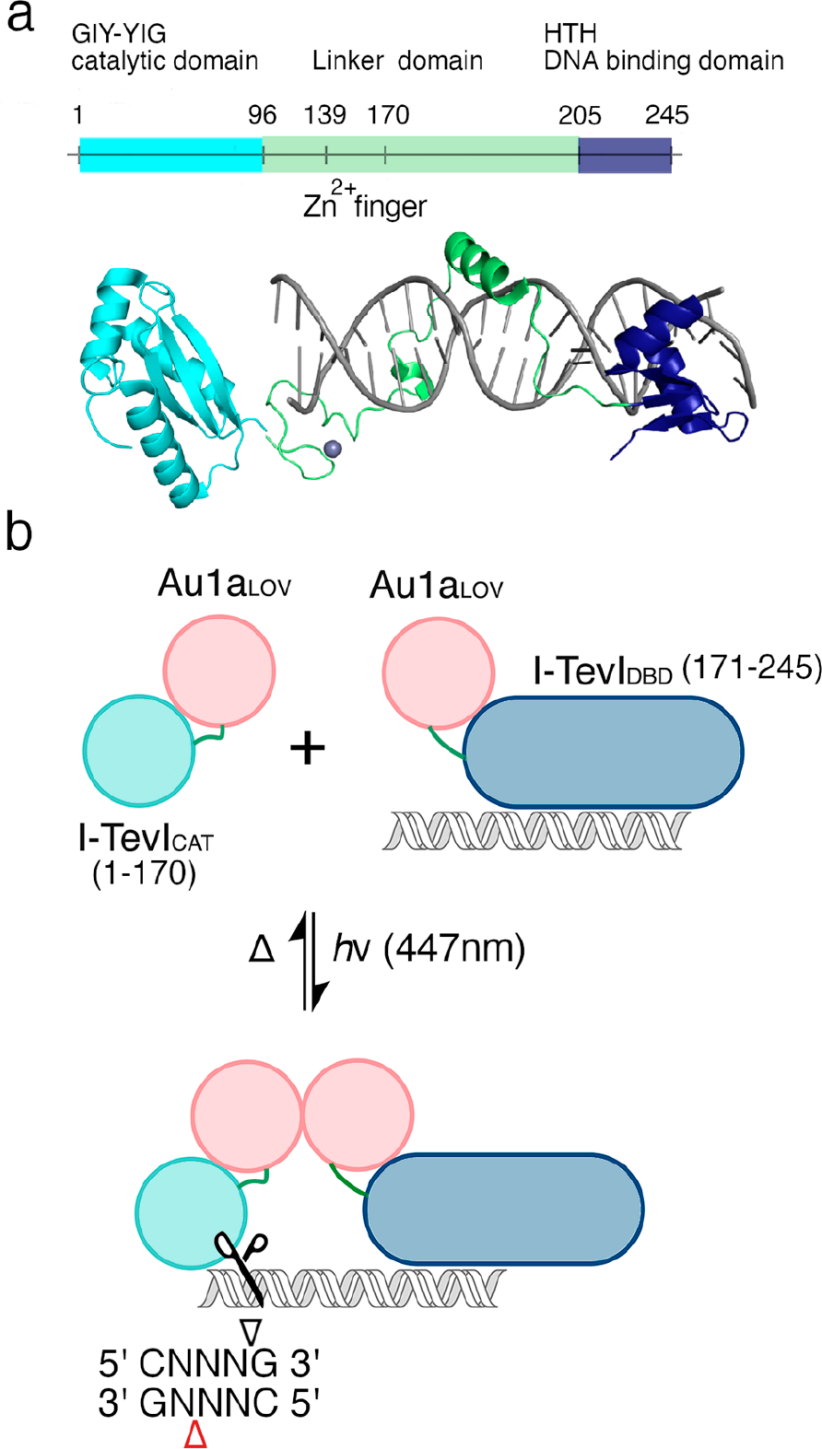
Design of I-TevI homing endonuclease system for optical control of DNA cleavage. (a) Structure of I-TevI homing endonuclease, comprising N-terminal GIY-YIG nuclease domain (light blue, PDB: 1LN0, residues 1–96) and C-terminal HTH DNA binding domain (navy, PDB 1T2T, residues 205–245). The connecting linker domain (green, PDB 1T2T residues 97–204) contains a ZF motif (residues 139–170). (b) The I-TevI light-induced nuclease is based on splitting the monomeric I-TevI homing endonuclease into two polypeptides, one comprising the nuclease domain (light blue) and the other the HTH DBD (navy) and both fused to an Au1a LOV domain (red). Under blue light conditions, dimerization of the I-TevI catalytic and DBD parts through Au1a domains was designed to increase cleavage of the homing site. Red and black triangles describe top and bottom strand nicking sites within the 5′ CNNNG 3′ cleavage motif.

The I-TevI homing endonuclease is encoded within the thymidylate synthase gene of bacteriophage T4.^27,28^ It has been developed into a programmable endonuclease for genome editing.^17,19,20,29^ Its modular domain arrangement and activity as a monomer has made it ideal for controlling site-specific dsDNA cleavage. I-TevI is composed of a N-terminal GIY-YIG^30,31^ nuclease domain and C-terminal helix-turn-helix (HTH) DNA binding domain (DBD) joined by a flexible linker (Figure 1a).^32,33^ The I-TevI nuclease recognizes a 38 bp homing site with nanomolar affinity, the specificity of which is dictated by contacts made through the flexible linker and HTH DBD.^32−35^ The GIY-YIG nuclease domain cleaves both strands of the target DNA at its minimal 5′-CN↑NN↓G-3′ cleavage motif to leave two nucleotide overhangs, while a ZF motif positioned within the linker region acts as a molecular “ruler” positioning the nuclease domain 28 bp upstream from the DBD binding site.^33^ Crystal structures show that the flexible linker binds to the minor groove of the target DNA, permitting a degree of sequence tolerance within the homing site (Figure 1a).^34−37^ Replacing the natural HTH DBD domain of I-TevI with ZFs, TALEs, or alternative inactive homing endonucleases has enabled reprogramming of sequence specificity for targeted genome editing.^17,29,38^ For these programmable I-TevI endonucleases, there is a strict distance requirement between the catalytic domain and DBD binding sites on the target DNA. This distance constraint has been exploited in I-TevI Cas9 chimeras to cut dsDNA at two locations and give defined deletion lengths to improve methods for nonhomologous end joining gene knockouts.^19^ Here the modular domain arrangement of I-TevI was adapted to create proteins where light-induced dimerization controls DNA cleavage. Partitioning DNA binding and endonuclease activities into different polypeptides allows for control through light-oxygen-voltage (LOV) photoreceptors with the potential to manipulate catalytic properties and cleavage precision by adjusting duration, distribution, and intensity of the irradiation.

LOV domain photoreceptors are versatile proteins for engineering photoresponsive biological systems due to their small size and their well-established and reversible mechanism of optical control over both natural and artificial effector modules.^39^ LOV photoreceptors utilize a flavin chromophore, which through blue light irradiation reacts with a conserved cysteine to form a semistable cysteinyl-flavin adduct. Signal transduction from the chromophore-binding pocket to effector modules occurs through N- and C-terminal helices^40^ and is mediated by the central β-sheet of the LOV domain core.^41,42^ NMR measurements have suggested that a thermodynamic driving force of ∼3.8 kcal mol^–1^ of free energy is available for light-driven effector activation,^43^ offering a dynamic range of more than 100-fold between the dark and light (photoactivated) states. Examples such as the LovTAP transcription factor based on *Avena sativa* LOV2 have shown that from originally modest responses improvements of the dynamic range can be achieved by rational engineering.^44,45^ Previously, we have shown that *Ochromonas danica* Aureochrome 1a (Au1a) homodimerizes in response to irradiation with blue light; the light state relaxes with a half-life of 112 min.^46^ Here we exploit this LOV photoreceptor in an artificial light-induced dimerization system to control homing nuclease activity with blue light for transient activation and a modular structure to program sequence specificity.

## Results and Discussion

Chimeric fusions of the GIY-YIG nuclease domain and HTH DBD of I-TevI with the Au1a LOV domain were constructed to separate the DNA cleavage and binding activities of I-TevI (Figure 1a,b). For previously engineered endonucleases, the I-TevI nuclease was truncated between residues 169 and 206 of the linker domain to use alternative DBDs to control target specificity.^17,29^ Notably, the ZF motif (residues 139–169) is essential for GIY-YIG catalytic activity.^32^ Here, for optical control, the I-TevI nuclease was split at residue 170 directly adjacent to the ZF to minimize any affinity the nuclease domain could have for DNA in the absence of the native I-TevI DBD (Figure 1). Specificity toward the minimal 5′--CN↑NN↓G-3′ cleavage site through ZF and nuclease specific contacts was designed to be retained. The I-TevI GIY-YIG nuclease domain (residues 1–170) was fused to the N-terminus of the Au1a LOV domain, generating construct I-TevI_CAT_(1–170)–Au1a, while the linker and HTH domains (residues 171–245) were fused to the C-terminus of Au1a LOV domain, forming construct Au1a–I-TevI_DBD_ (Supporting Information). This split design reduced the toxicity of the nuclease to *E. coli*, enabling plasmid assembly whereas the native full-length I-TevI sequence is intractable to cloning and expression.^47^

**Figure 2:**
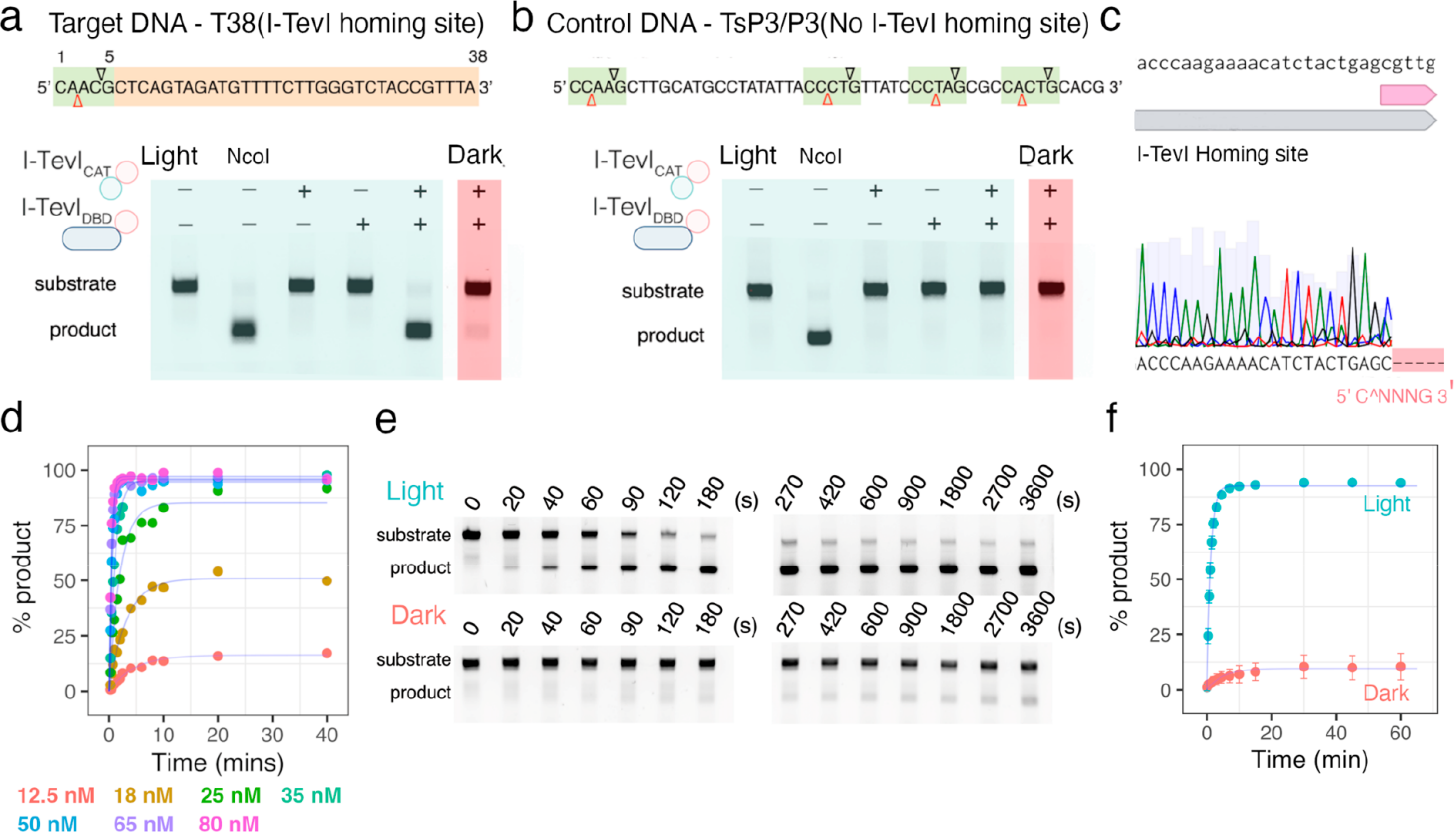
Characterization of the split I-TevI Au1a endonuclease system. (a) Cleavage of cy5.5-labeled T38 target DNA with homing site with different combinations of parts (I-TevI_CAT_(1–170)–Au1a and Au1a–I-TevI_DBD_) under blue light and dark conditions. (b) Control cleavage reaction of cy5.5-labeled TsP3/P3 DNA lacking I-TevI homing site. Several CNNNG cleavage sites are present but not cut. (c) Sequencing of the product of the T38 target DNA after cleavage under blue light. (d) Cleavage reaction with different concentrations (12.5–80 nM) of a 1:1 molar ratio of I-TevI_CAT_(1–170)–Au1a and Au1a–I-TevI_DBD_, and 13 nM substrate. (e) Single-turnover cleavage of T38 DNA with 1:1 mixed I-TevI catalytic and DBD parts. f) Plot of single-turnover cleavage activity under blue light and dark conditions, plotted as means ± SD, *n* = 3.

The light-induced dimerization of nuclease and DNA binding parts was designed to enhance affinity of the nuclease domain for its target DNA over monomeric components and thereby result in DNA cleavage under blue light conditions (Figure 1b). The nuclease activity of I-TevI_CAT_(1–170)–Au1a and Au1a–I-TevI_DBD_ constructs were tested both independently and combined under dark (performed under low intensity red light, 623 nm) and illuminated conditions (450 nm). Two ∼600 bp cyanine 5.5-labeled fluorescent DNA substrates were used, T38 containing the complete 38 bp I-TevI homing site (Figure 2a) and TsP3/P3, which lacks the inserted homing site and internal NcoI control cleavage site (Figure 2b). Although TsP3/P3 does not have the homing site, the minimal catalytic cleavage CNNNG motif is present due to its high frequency of occurrence. For both target DNAs, when I-TevI_CAT_(1–170)–Au1a and Au1a–I-TevI_DBD_ were incubated independently for 2 min no cleavage products were observed (Figure 2a,b). Only on mixing both I-TevI_CAT_(1–170)–Au1a and Au1a–I-TevI_DBD_ in a 1:1 molar ratio under blue light (450 nm) was 95% of the T38 substrate cut. In dark conditions, cleavage of the T38 substrate was significantly reduced to 13%. The product has a well-defined band on 2% agarose gel, equivalent to control digestions of restriction enzyme NcoI and for which the cleavage site is adjacent to the I-TevI homing site. Sequencing of the fluorescently labeled product mapped the cleavage site to the anticipated CNNNG motif of the T38 homing site (Figure 2c). The TsP3/P3 control substrate lacking the homing site was not digested under light or dark conditions. Likewise, the activity for three alternative substrates containing truncated homing site sequences (T33, T27, and T23) was reduced compared to the full-length homing substrate (Figure S1). Taken together, these data confirmed the achievement of our initial design goal and that the light-induced dimerization of I-TevI_CAT_(1–170)–Au1a and Au1a–I-TevI_DBD_ through the Au1a LOV domain enhances catalysis over the activity of the isolated components containing only either the GIY-YIG or HTH domains of I-TevI.

Next, single-turnover cleavage assays following nuclease activity under dark and illuminated conditions were performed for I-TevI_CAT_(1–170)–Au1a and Au1a–I-TevI_DBD_ parts mixed in a 1:1 ratio to establish the kinetic behavior of DNA cleavage (Figure 2d–f). Catalysis of I-TevI has been described previously to have an initial burst phase, followed by a slower steady-state rate likely due to product inhibition.^29^ Our Au1a split system displayed a similar single-turnover profile under both light and dark conditions (Figure 2d–f). By varying the enzyme concentration under blue light (Figure 2d), it was apparent that the initial single-turnover rate (*k*_obs_) dictated the catalytic turnover for 40 min (Figure 2d). After the initial exponential turnover no further cleavage was observed most likely due to product inhibition.^29^ Using 125 nM I-TevI_CAT_(1–170)–Au1a and 125 nM I-TevI_DBD_ with 13 nM substrate DNA, a 4-fold difference in *k*_obs_ between illuminated (*k*_obs_(light), 0.87 ± 0.02 min^–1^) and dark-state reactions (*k*_obs_(dark), 0.21 ± 0.04 min^–1^) was observed (Figure 2f). This corresponds to a ∼7-fold reduction in activity of the light-induced dimerization system under blue light when compared with previously reported monomeric I-TevI constructs, most likely due to unoptimized steric constraints from the chimeric fusion to the LOV domain and reduced affinity for DNA.^29^ The kinetic profile for the dark-state enzyme closely followed that observed for the equivalent light state with lower enzyme concentrations. This suggests that in the dark, a lower active concentration of functional nuclease is present due to the difference in dimerization affinities of the LOV domain under dark and blue light conditions. At these concentrations, target DNA is almost entirely cleaved under blue light illumination, with only 10% cleavage after 60 min in the dark, equating to a 9-fold difference in yield of cleavage products. It should be noted that when tested at 10-fold higher enzyme concentrations (1.25 μM), significant off-target nuclease activity was observed and TevI_CAT_(1–170)–Au1a was active in the absence of the Au1a–I-TevI_DBD_ construct (Figure S2), illustrating how balancing the affinities and concentrations of the DNA and I-TevI parts is essential for obtaining optical control over activity and specificity.

**Figure 3:**
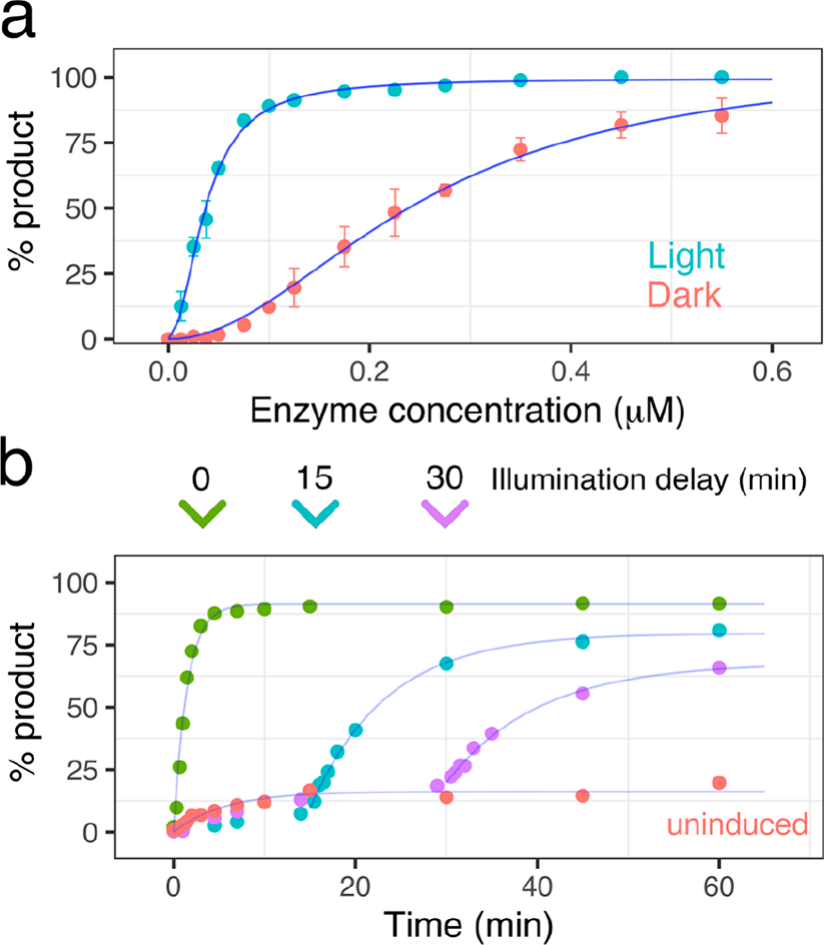
Demonstration of the light-induced dimerization I-TevI homing endonuclease system. (a) Plot following the effect of increasing enzyme concentration on the dynamic range. Plotted as means ± SD, *n* = 3 and fitted with a Hill-coefficient of 2 due to cooperativity between catalytic and DBD (I-TevI_CAT_(1–170)–Au1a and Au1a–I-TevI_DBD_). EC_50_ values of 0.04 μM and 0.25 μM were determined for light and dark conditions, a 6-fold difference in activity. (b) Graph of temporal control of activity by inducing DNA cleavage with blue light at time points of 0, 15, and 30 min as well as an uninduced control reaction.

To better understand how the enzyme concentration altered the optical response of the light activated system, the percentage cleavage arising from the initial turnover was measured under illuminated and dark conditions for a range of enzyme concentrations and 13 nM substrate (Figure 3a). Both I-TevI_CAT_(1–170)–Au1a and Au1a–I-TevI_DBD_ were held in a 1:1 molar ratio. A sigmoidal cleavage curve was observed and fitted using a Hill coefficient of 2, which is consistent with cooperativity between the I-TevI_CAT_(1–170)–Au1a and Au1a–I-TevI_DBD_ constructs. The enzyme concentration that resulted in 50% cleavage under illuminated and dark conditions (EC_50_) was 0.04 ± 0.01 μM and 0.25 ± 0.07 μM, respectively, equating to a 6-fold difference in endonuclease activity between light and dark states, a noteworthy level of optical control for a first-generation design and likely to be in part due to the utility of Au1a LOV domain for engineering. To further demonstrate the potential of the split I-TevI homing endonuclease system for temporal control, the endonuclease activity was induced after 15- and 30 min using blue light (Figure 3b). An increase in activity was observed upon illumination in both cases, and a single exponential was fitted after the point of induction. The single-turnover rate and ultimate yield after 60 min for each delayed reaction (15, 30 min inductions) was reduced compared with the reaction with no delay (0 min), possibly due to instability of the catalytic domain, which was observed to precipitate at higher concentrations under reaction conditions. Nonetheless, a clear enhancement of catalysis occurs with blue light and demonstrates the potential for temporal control for DNA editing methods.

**Figure 4:**
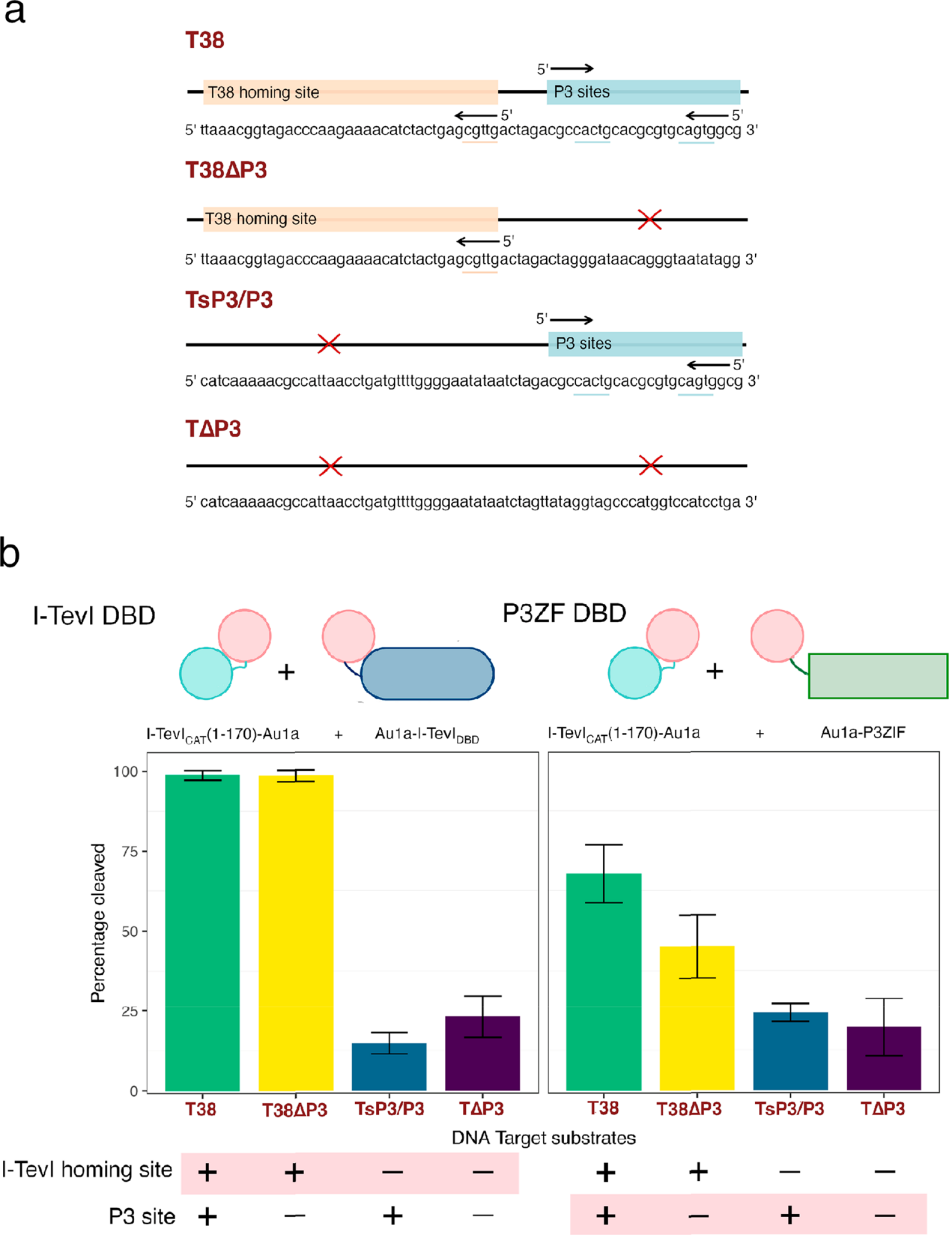
Comparison of substrate preference with different DBD modules. (a) Illustration of four DNA substrates assembled with or without the T38 homing site and/or palindromic P3 target sites. Target CNNNG sites are underlined. Deleted sequences are indicated with a red cross (b) Left: (Native I-TevI DBD, Au1a–I-TevI_DBD_), right: (P3ZF DBD, Au1a–P3ZF) in a 1:1 molar ratio with the I-TevI_CAT_(1–170)–Au1a nuclease. Substrates have all possible combinations of the I-TevI homing and sP3/P3 sites as detailed with the positive and negative signs. Highlighted sites with positive sign are the substrates that are predicted to undergo cleavage with the respective DBD parts. Intrinsic specificity of the I-TevI_CAT_(1–170)–Au1a nuclease part for the I-TevI homing site means cleavage of T38 and T38ΔP3 is favored in both cases. Data are plotted as means ± SD, *n* = 3.

Next, the modular capacity of the split Au1a I-TevI homing nuclease system was investigated to establish whether the DNA target sequence could be reprogrammed by exchanging the DBD module. A new DBD construct was prepared comprising the Au1a LOV domain fused to the tandem zinc finger domain, P3ZF (Au1a–P3ZF, Supporting Information). The DNA recognition site of the P3ZF, 5′-GCA GTG GCG-3′, is significantly shorter than that of the native HTH and linker domains of I-TevI. Four different substrates containing different combinations of the I-TevI homing site and a palindromic sP3/P3 zinc finger site (Figure 4a,b) were compared by single point cleavage experiments using the I-TevI_CAT_(1–170)–Au1a nuclease. By exploiting a palindromic sP3/P3 sequence (TsP3/P3), we aimed to target the internal 5′-CACTG-3′ cleavage site within the P3 binding site rather than the previously used native I-TevI sequence. The total target length of the sP3/P3 target was 18bp, and two binding and cleavage modes were anticipated (Figure 4a, Supporting Information). With the native I-TevI DBD, Au1a–I-TevI_DBD_, cleavage was observed for substrates that contained the cognate homing site (T38, T38ΔP3) but not the substrates lacking the homing site (TsP3/P3, TΔP3) (Figure 4b). With the alternative Au1a–P3ZF DBD there were changes in sequence preference but the substrates containing the I-TevI homing site were still preferentially cleaved over substrates with the P3 sites. It is likely that P3ZF DBD was unable to fully alter the specificity of the split I-TevI endonuclease system because I-TevI_CAT_(1–170)–Au1a retained some specificity for the native homing site. The source of this affinity was expected to arise through the nuclease ZF motif (residues 130–169), which is known to be important in maintaining the distance constraints for cleavage of the full-length nuclease and which was retained in the split nuclease domain. Therefore, to probe the effect of the I-TevI ZF on the promiscuity of the nuclease domain, the I-TevI_CAT_(1–130)–Au1a catalytic construct was generated without the native I-TevI ZF motif (Supporting Information).

**Figure 5:**
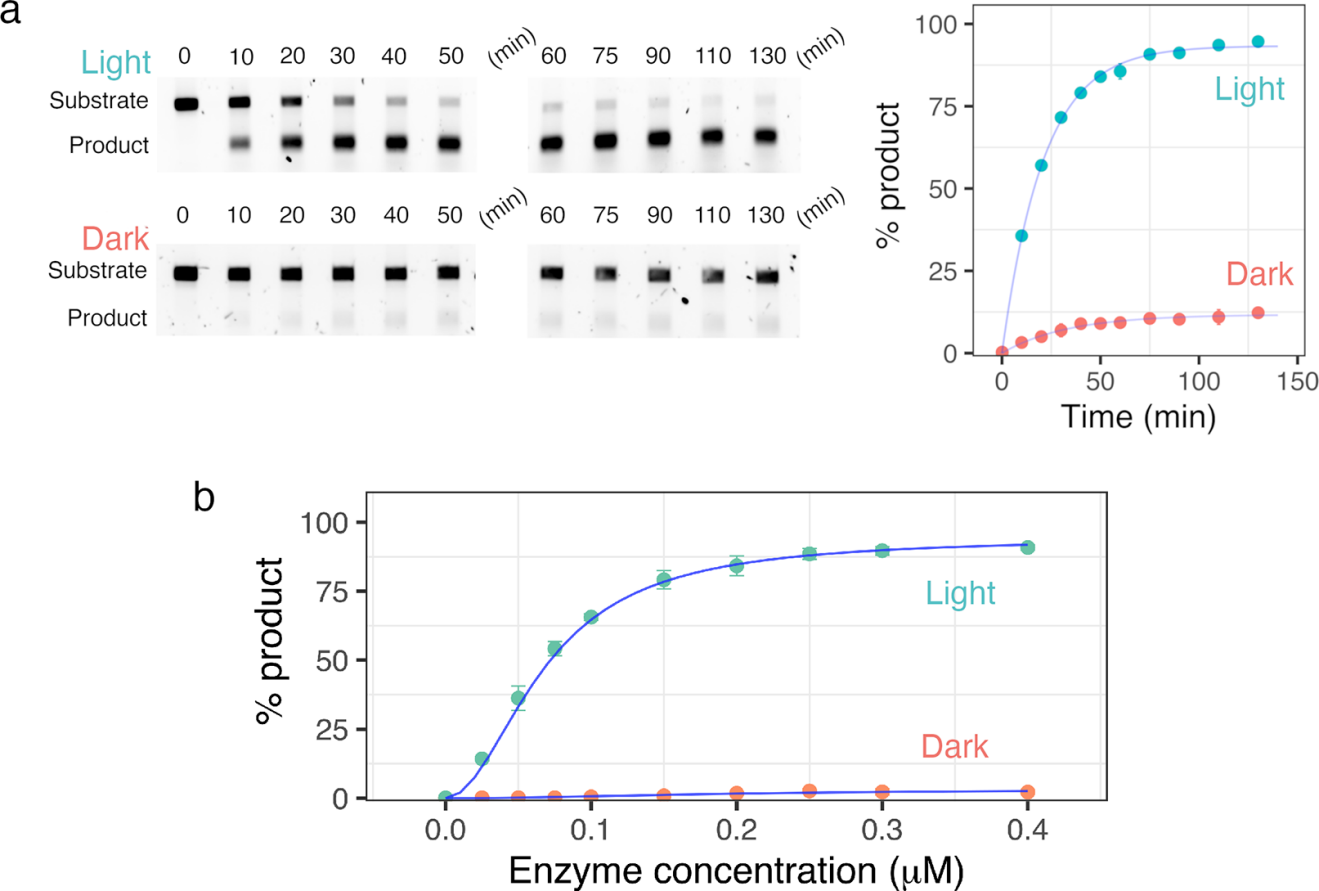
Kinetic characterization of the truncated nuclease part, I-TevI_CAT_(1–130)–Au1a with Au1a–I-TevI_DBD_ for the I-TevI homing site under blue light and dark conditions. (a) 2% agarose DNA gel following cleavage of cy5.5 T38 substrate and plot of percentage cleavage taken at each time point with T38 substrate. (b) Cleavage of T38 substrate at increasing enzyme concentrations. Nuclease and DBD parts were held in a 1:1 molar ratio and fitted with a Hill-coefficient of 2. EC_50_ of 0.07 μM under light conditions. Data are plotted as means ± SD, *n* = 3.

Single-turnover cleavage assays for equimolar mixtures (230 nM) of I-TevI_CAT_(1–130)–Au1a and Au1a–I-TevI_DBD_ parts were performed for the native I-TevI homing site T38 substrate (13 nM) (Figure 5a,b). Similar kinetic behavior was observed for the truncated nuclease under blue light and dark conditions, giving rate constants of *k*_obs_(light) of 0.05 min^–1^ and *k*_obs_(dark) of 0.03 min^–1^, respectively. As anticipated, removing the I-TevI nuclease ZF motif reduced the enzyme activity, likely because of the reduced affinity of the nuclease domain for the 5′-CNNNG-3′ nuclease site. Nonetheless, optical control of the nuclease activity was retained, resulting in near full cleavage of the I-TevI homing site under blue light after 2 h, while in the dark cleavage was limited, yielding an 8-fold difference in final cleavage. Nonspecific products were observed for the truncated I-TevI_CAT_(1–130)–Au1a nuclease in both dark and illuminated reactions, and there was an overall loss of fluorescence intensity across the time course, which had not previously been observed for the I-TevI_CAT_(1–170)–Au1a construct (Figure S3). It was clear that removal of the ZF motif reduced cleavage fidelity of the homing site irrespective of illumination conditions. An EC_50_ of 0.07 ± 0.01 μM under illuminated conditions was established by varying the concentration of I-TevI_CAT_(1–130)–Au1a and Au1a–I-TevI_DBD_ relative to the T38 homing site substrate (Figure 5b), comparable to that for complexes containing the more active I-TevI_CAT_(1–170)–Au1a. This demonstrates that although the initial rate is reduced, the enzyme concentration required to cleave a given concentration of DNA is maintained. An accurate concentration of nuclease that results in 50% cleavage (EC_50_) could not be determined for the dark state because enzyme concentrations above 0.4 μM led to significant nonspecific cleavage for both the light and dark conditions due to the lesser specificity of the construct lacking the I-TevI ZF. Nonspecific cleavage of DNA is the dominant activity in the dark without dimerization of I-TevI_CAT_(1–130)–Au1a and Au1a–I-TevI_DBD_.

**Figure 6:**
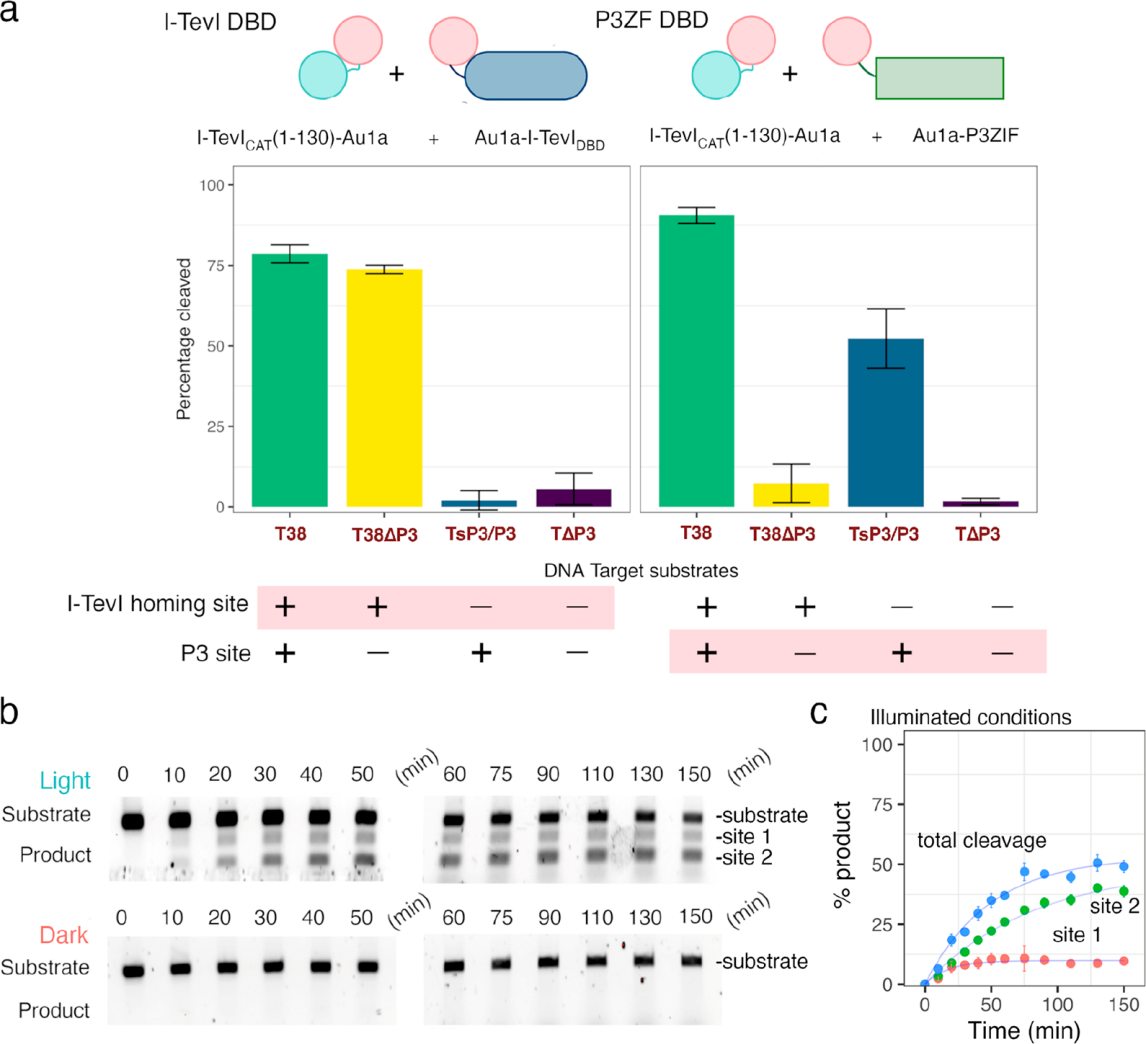
Substrate preference of truncated nuclease with altered DBD modules, (a) Left: (native I-TevI DBD, Au1a–I-TevI_DBD_), right: (P3ZF DBD, Au1a–P3ZF) in a 1:1 molar ratio with the I-TevI_CAT_(1–130)–Au1a nuclease. (b) 1.5% agarose DNA gel following cleavage of cy5.5 TsP3/P3 substrate with I-TevI_CAT_(1–130)–Au1a and Au1a–P3ZF parts in a 1:1 ratio. Two cleavage sites are observed. (c) Plot of the relative fluorescent intensities for site 1, site 2, and the total cleavage for the observed single-turnover cleavage under blue light. No significant cleavage under dark conditions was observed. Data are plotted as means ± SD, *n* = 3.

To establish whether I-TevI_CAT_(1–130)–Au1a could be reprogrammed to target the P3 site using the Au1a–P3ZF DBD, the substrate preference was examined by single point cleavage experiments with 2 h incubation periods for four substrates with different combinations of the I-TevI homing and P3 target sites (Figure 6a). Under blue light, the substrate preference using both Au1a–I-TevI_DBD_ and Au1a–P3ZF DBD were compared. As for the more active I-TevI_CAT_(1–170)–Au1a construct, the truncated I-TevI_CAT_(1–130)–Au1a nuclease displayed preference for the native I-TevI homing site (substrates T38, T38ΔP3) with the corresponding Au1a–I-TevI_DBD_. Exchanging the DBD partner for Au1a–P3ZF resulted in a change in substrate preference of the I-TevI_CAT_(1–130)–Au1a nuclease for substrates with the P3 sites (T38, TsP3/P3). Single-turnover cleavage kinetics were measured for the TsP3/P3 substrate under blue light and in the dark with I-TevI_CAT_(1–130)–Au1a and Au1a–P3ZF in a 1:1 ratio (Figure 6b,c). Cleavage of the TsP3/P3 substrate occurred under illuminated conditions and resulted in two product bands defined as site 1 and site 2. On review of the target DNA, a second P3-like binding site differing in a single nucleotide (5′ GCA GTA GCG 3′) was found 126 bp downstream of the designed site. As numerous minimal CNNNG cleavage sites are present, this site is likely to give rise to the second cleavage band detected. As previously observed for the I-TevI_CAT_(1–130)–Au1a construct with Au1a–I-TevI_DBD_, significant nonspecific cleavage was observed which impeded sequencing of the cut sites and resulted in a reduction of ∼50% fluorescence intensity for kinetic experiments under both dark and light conditions. No cleavage was observed under dark conditions. The two cleavage sites highlight how, for monomeric nucleases, the 9 bp ZF target site is not specific enough for genome editing applications, whereas for applied ZFNs, dimerization of the nuclease domain doubles target length to overcome this limitation. Taken together, these data demonstrate that removal of the intrinsic ZF motif from the I-TevI nuclease module allows the reprogramming of substrate selectivity, albeit at a cost in overall catalytic activity and fidelity.

The native I-TevI nuclease is toxic to *E. coli*.^47^ To determine if this is true of the split nuclease system when coexpressed, the of I-TevI_CAT_(1–170)–Au1a and Au1a–I-TevI_DBD_ parts were assembled into a plasmid in a single operon under the rhamnose promoter and transformed into *E. coli*. Coexpression of the nuclease parts with 0.3% and 1% rhamnose under blue light significantly reduced the survival rate compared with the unexpressed controls in plasmids with and without the additional RhaS gene (rhamnose promoter activator), Figure S4. Like the native I-TevI nuclease, this confirms that the split system cleaves genomic dsDNA in vivo but will require the nuclease fidelity and programmable sequence specificity to be further optimized for applications in *E. coli* and other organisms to limit any potentially constraining toxicity.

## Conclusion

Here we report the design, synthesis, and characterization of a novel modular photoresponsive nuclease comprising components of the monomeric I-TevI homing endonuclease split into different polypeptides and fused with the Au1a LOV domain in a light-induced dimerization system. The Au1a LOV photoreceptor proved a facile and rapid protein system for establishing control by blue light irradiation with minimal design challenges; for the first time, a dynamic range of 6- to 9-fold was achieved even without optimization. The light-induced dimerization system reported here can be programmed to target alternative DNA sequences through exploiting different DBDs as for monomeric I-TevI endonucleases,^17,29,38^ but this comes at the cost of having to reduce overall nuclease activity of the I-TevI nuclease by removing the internal ZF motif which in turn reduced cleavage fidelity. Numerous robust in vivo selection screens have been developed for improving gene-editing nucleases for application and can now be exploited to increase activity and improve fidelity.^7,16,48,49^ Our system opens the possibility for a library of light controlled nucleases with alternative DBDs to the I-TevI HTH and P3ZF used here for programmed DNA cleavage including using the inactive variant of CRISPR-dCas9 and guide RNAs to drive cleavage.^19,50^ For application, the use of a light-induced dimerization homing endonuclease system requires balancing the affinities between nuclease and DBD modules, each module for the DNA, and catalytic activity. Likewise, an appropriate time frame for cleavage must be established to ensure that the reduced activity in the dark does not reach light-state levels. With this approach, engineering modern genetic tools with optical control is now readily obtainable. For gene-editing applications where cleavage precision is paramount, higher fidelity is first required before the photoresponsive I-TevI nuclease can be fully exploited. The modular I-TevI nuclease is however well suited for *in vitro* purposes and may have applications for controlling the top-down synthesis of DNA nanostructures for 2D and 3D DNA architectures and microarrays and the release of molecular payloads from DNA-based precursors.^51,52^ As these applications typically do not require high fidelity, the light-responsive I-TevI nuclease developed here could be applied without further engineering for optical control. In summary, we have demonstrated that monomeric homing endonucleases can be reengineered for optical control with programmable specificity by fusion of independent functional domains to LOV domain photoreceptors with notable dynamic range for a first-generation platform.

## Methods

### Plasmid Assembly

To construct plasmids encoding I-TevI_CAT_(1–170)–Au1a, Au1a–I-TevI_DBD_, and Au1a–P3ZF, fragments of I-TevI, Au1a, and P3ZF genes were amplified by PCR using oligonucleotides detailed in Table S1 and PrimeStarHS (Takara Bio. Inc.). Plasmid templates for Au1a were kindly provided by Prof. Harald Janovjak (Flinders University), for P3ZF (pPDAZ.P3-Sharkey) by Prof. Carlos F. Barbas, III (The Scripps Research Institute),^16^ and for I-TevI purchased from GenScript in vector PCCI. PCR products were gel purified and assembled into a modified pET28a vector with N-terminal His_6_ tag and TEV protease site using Golden Gate assembly methods.^53^ The truncated I-TevI_CAT_(1–130)–Au1a was constructed by blunt-end ligation from the larger I-TevI_CAT_(1–170)–Au1a_LOV_ plasmid using oligonucleotides detailed in Table S1 and the KLD kit (NEB). All constructs were sequenced (Eurofins Genomics) to confirm correct assembly from the T7 promotor and sequences are available in the Supporting Information.

Plasmids containing the target DNA sites for endonuclease cleavage were constructed by restriction digestion and ligation methods. The original TsP3/P3 plasmid was kindly provided by Prof. Carlos F. Barbas, III (The Scripps Research Institute).^16^ To this plasmid was added the I-TevI endonuclease homing site (T38) by annealing two oligonucleotides (Table S1) with SpeI sites at both ends, digesting with SpeI, and ligating within the XbaI site of the TsP3/P3 plasmid using XbaI and T4 ligase (NEB). Constructs with a single homing site addition were confirmed by sequencing using the sequencing oligonucleotide detailed in Table S1. The TΔP3 control plasmid and truncated homing endonuclease site plasmids (T23, T27, and T33) were made using the same methods with alternative oligonucleotides (Table S1). The plasmid T38ΔP3 where the P3 sites were removed was generated from T38 plasmid by blunt-end ligation and oligonucleotides detailed in Table S1.

### Protein Expression and Purification

Plasmids encoding I-TevI_CAT_(1–170)–Au1a, I-TevI_CAT_(1–130)–Au1a, Au1a–I-TevI_DBD_, and Au1a–P3ZF were freshly transformed for each expression into the BL21(AI) *E*. *coli* strain and plated on LB agar supplemented with 1% glucose and 50 μg mL^–1^ kanamycin. Colonies were picked and grown for 16 h in LB media with 1% glucose and 50 μg mL^–1^ kanamycin at 37 °C. Five milliliters of the initial culture was added to 500 mL of terrific broth media supplemented with additional 1% glucose and 50 μg mL^–1^ kanamycin at 37 °C and shaking at 250 rpm in the dark. Large-scale cultures were grown until OD_600 nm_ 1.0, and protein production was induced by addition of 4 g L^–1^l-arabinose. Cultures were grown for a further 16 h at 16 °C before cells were harvested by centrifugation at 6000 rpm for 15 min and pellets frozen at −20 °C.

All proteins were purified by immobilized metal affinity chromatography (IMAC) under red light (623 nm, 14.4W 24 V SMD 5050 red LEDs, Ledxon Modular) to ensure that the Au1a LOV domain was maintained in the dark state before further application. Cell pellets were suspended in lysis buffer, 50 mM HEPES, 300 mM NaCl, 1 mM TCEP with 5 mg of lysozyme, and 10 mg of PMSF and sonicated at 4 °C. The insoluble cell debris was removed by centrifugation at 16 000 rpm for 40 min. The supernatant was applied to a Ni^2+^-NTA column equilibrated in buffer and a gradient of imidazole (20, 40, 60, 250 mM) containing lysis buffer applied. Once purified, the imidazole was removed by buffer exchange into lysis buffer, and TEV protease was used to remove the His_6_ tag by overnight proteolysis at 4 °C. The I-TevI_CAT_–Au1a constructs required large amounts of TEV protease, equating to 0.2 equiv in order to completely remove the His_6_ tag, possibly due to steric hindrance through its N-terminal location in the I-TevI catalytic domain. After cleavage was complete, proteins were passed through a Ni^2+^-NTA column in lysis buffer supplemented with 20 mM imidazole. An additional 2 mg of FMN was added to ensure saturation of the LOV photoreceptor, and the protein buffer was exchanged with lysis buffer, concentrated to 250 μM, and stored at −20 °C. An extinction coefficient for Au1a of 9135 M^–1^ cm^–1^ under fully illuminated (blue light, 450 nm) conditions was used to establish protein concentration. UV spectral changes (200–600 nm) between light and dark conditions confirmed that the blue light LEDs used were sufficiently bright to fully switch all proteins used with <20 s of exposure.

DNA cleavage assays: Fluorescent substrate DNA was generated by PCR amplification using PrimeStarHS (Takara Bio. Inc.) and T38, T38ΔP3, TsP3/P3, or TΔP3 templates using primers detailed in Table S1, giving 545–622 bp fragments labeled at the 5′ end of the forward strand with cyanine 5.5 fluorophore. Fragments were gel purified on a 1.5% agarose gel in TAE buffer and eluted in H_2_O at concentrations between 50 and 200 ng μL^–1^. Target substrates were analyzed using a nanodrop spectrophotometer (ThermoFisher Scientific) to ensure reproducibility between substrate batches and the quality of DNA isolated.

DNA cleavage reactions were set up in CutSmart buffer (50 mM potassium acetate, 10 mM magnesium acetate, 100 μg/mL BSA, pH 7.9) supplemented with 1 mM dithiothreitol (DTT). Nuclease and DBD proteins were diluted to 1.25–2.5 μM and the different constructs mixed in a 1:1 ratio or kept separate to generate stocks in CutSmart buffer supplemented with 1 mM DTT. Fluorescent target DNAs were diluted to 5 ng μL^–1^, and single time point (2 min I-TevI_CAT_(1–170)–Au1a, 2 h I-TevI_CAT_(1–130)–Au1a) cleavage experiments were performed under red light (623 nm, 24 V SMD 5050 red LEDs) or blue light (450 nm, 12 V SMD 5050 blue LEDs) using 0.125 μM enzyme. Reactions were quenched using gel loading dye, Purple (NEB) containing SDS, and boiled at 80 °C for 5 min. Samples were analyzed using 1.5–2% agarose gel in TAE buffer and visualized using a ChemiDoc MP Imaging system (BioRad) using a far red epi illumination source and 715/30 emission filter to observe the cyanine 5.5 fluorophore. Agarose gels were analyzed using Image Lab 6.1 (BioRad) with lanes manually defined and bands selected using automated detection. Intensities were analyzed as a percentage of the overall intensity of detected bands within each lane to ensure that any loading differences between lanes did not lead to inflated errors.

Single turnover time course reactions were performed in triplicate under dark (red light, 623 nm) and light conditions (blue light, 450 nm). Large-scale reactions (200–600 μL) with 5 ng μL^–1^ fluorescent target DNA with 0.0625–0.240 μM endonuclease (either I-TevI_CAT_(1–170)–Au1a or TevI_CAT_(1–130)–Au1a) and DBD (either Au1a–I-TevI_DBD_ or Au1a–P3ZF) in a 1:1 ratio in CutSmart buffer plus 1 mM DTT were generated. Twenty microliter aliquots of the reaction were quenched at defined time points with 4 μL of gel loading dye, Purple (NEB), and samples were boiled at 80 °C for 5 min before being analyzed with a 1.5–2% agarose gel. Reaction kinetics were fitted to the equation
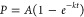
where *P* is percentage cleavage, *A* is the maximum cleavage, *k* is the observed rate, and *t* is time.

To establish the concentration of nuclease required for 50% cleavage (EC_50_) under dark (red light) and light (blue light) conditions, the enzyme concentration was varied between (12.5 nM to 0.55 μM) and the percentage of cleavage was established as defined for single turnover reactions for a single time point of 2 min for I-TevI_CAT_(1–170)–Au1a and 130 min for the less active TevI_CAT_(1–130)–Au1a construct. EC_50_ was determined by fitting to the equation
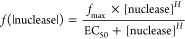
where *f*(|nuclease|) is the fraction of fluorescent substrate cleaved for any given nuclease concentration, *f*_max_ is the maximum cleavage observed, and [nuclease] is the nuclease concentration used. A Hill coefficient, *H*, of 2 was determined to be most suitable for the system demonstrating cooperativity between catalytic and DBD constructs.

Single point cleavage reactions to compare nuclease substrate preference when exchanging DBDs were performed with a 2 min digestion for 0.125 μM I-TevI_CAT_(1–170)–Au1a and 120 min digestion for 0.230 μM TevI_CAT_(1–130)–Au1a construct in CutSmart buffer plus 1 mM DTT. Each fluorescent substrate (T38, T38ΔP3, TsP3/P3, TΔP3) was diluted to 5 ng μL^–1^ and the percentage cleavage determined for each DBD held in a 1:1 ratio with the nuclease domain from a 2% agarose gel.

### DNA Cleavage Mapping

Sequencing of cleavage products was achieved by performing cleavage reactions for 1 μg of substrate DNA as outlined for single time point experiments. Products were gel extracted to an approximate concentration of 50 ng μL^–1^ in H_2_O and sequenced by Eurofins Genomics using the sequencing primer detailed in Table S1.

### Coexpression of TevICAT(1–170)–Au1a and Au1a–I-TevIDBD

Top10 *E. coli* chemically competent cells (100 μL aliquots) were transformed with 2 μL of 50 ng μL^–1^ plasmid DNA encoding the I-TevI_CAT_(1–170)–Au1a and Au1a–I-TevI_DBD_ parts under rhamnose promoter. Two plasmids were compared differing in whether the *E. coli* RhaS gene under the J23107 promoter was included on the plasmid. Cells were heat shocked at 42 °C for 45 s and placed on ice. One milliliter of SOB media was added, and cells were grown at 37 °C for 1 h. Rhamnose at the defined concentrations (0, 0.3% and 1%) was added, and cells were grown for a further 1 h under blue light. Cells were plated onto kanamycin LB agar and incubated for 16–20 h at 37 °C.

## Data Availability

Raw data files are available at https://doi.org/10.17035/d.2023.0297105866.

## Supporting Information Available

Additional figures for cleavage assays of target DNAs with truncated homing nuclease sites and nonspecific nuclease activity at high enzyme concentrations. Full construct DNA, protein, and oligonucleotide sequences (PDF)

## Abbreviations

HTH: helix-turn-helix motif
Au1a: Aureochrome1a
LOV: light-oxygen-voltage
ZFN: zinc finger nuclease
TALEN: transcription activator-like effector nucleases
CRISPR: clustered regularly interspaced short palindromic repeats
ZF: zinc finger
DBD: DNA binding domain

## References

[ref1] Chevalier B. S.; Stoddard B. L. Homing Endonucleases: Structural and Functional Insight into the Catalysts of Intron/Intein Mobility. Nucleic Acids Res. 2001, 29 (18), 3757–3774. 10.1093/nar/29.18.3757. 11557808 PMC55915

[ref2] Gimble F. S. Invasion of a Multitude of Genetic Niches by Mobile Endonuclease Genes. FEMS Microbiol Lett. 2000, 185 (2), 99–107. 10.1111/j.1574-6968.2000.tb09046.x. 10754232

[ref3] Beyer H. M.; Iwaï H. Structural Basis for the Propagation of Homing Endonuclease-Associated Inteins. Front Mol. Biosci 2022, 9, 855511 10.3389/fmolb.2022.855511. 35372505 PMC8966425

[ref4] Windbichler N.; Menichelli M.; Papathanos P. A.; Thyme S. B.; Li H.; Ulge U. Y.; Hovde B. T.; Baker D.; Monnat R. J.; Burt A.; Crisanti A. A Synthetic Homing Endonuclease-Based Gene Drive System in the Human Malaria Mosquito. Nature 2011, 473 (7346), 212–215. 10.1038/nature09937. 21508956 PMC3093433

[ref5] Verkuijl S. A. N.; Ang J. X. D.; Alphey L.; Bonsall M. B.; Anderson M. A. E. The Challenges in Developing Efficient and Robust Synthetic Homing Endonuclease Gene Drives. Front Bioeng Biotechnol 2022, 10, 856981 10.3389/fbioe.2022.856981. 35419354 PMC8996256

[ref6] Lu Y.; Happi Mbakam C.; Song B.; Bendavid E.; Tremblay J.-P. Improvements of Nuclease and Nickase Gene Modification Techniques for the Treatment of Genetic Diseases. Front Genome Ed 2022, 4, 892769 10.3389/fgeed.2022.892769. 35958050 PMC9360573

[ref7] Lee K. Z.; Mechikoff M. A.; Parasa M. K.; Rankin T. J.; Pandolfi P.; Fitzgerald K. S.; Hillman E. T.; Solomon K. V. Repurposing the Homing Endonuclease I-SceI for Positive Selection and Development of Gene-Editing Technologies. ACS Synth. Biol. 2022, 11 (1), 53–60. 10.1021/acssynbio.1c00340. 35007422

[ref8] Bibikova M.; Beumer K.; Trautman J. K.; Carroll D. Enhancing Gene Targeting with Designed Zinc Finger Nucleases. Science 2003, 300 (5620), 764–764. 10.1126/science.1079512. 12730594

[ref9] Kim Y. G.; Cha J.; Chandrasegaran S. Hybrid Restriction Enzymes: Zinc Finger Fusions to Fok I Cleavage Domain. Proc. Natl. Acad. Sci. U. S. A. 1996, 93 (3), 1156–1160. 10.1073/pnas.93.3.1156. 8577732 PMC40048

[ref10] Miller J. C.; Tan S.; Qiao G.; Barlow K. A.; Wang J.; Xia D. F.; Meng X.; Paschon D. E.; Leung E.; Hinkley S. J.; Dulay G. P.; Hua K. L.; Ankoudinova I.; Cost G. J.; Urnov F. D.; Zhang H. S.; Holmes M. C.; Zhang L.; Gregory P. D.; Rebar E. J. A TALE Nuclease Architecture for Efficient Genome Editing. Nat. Biotechnol. 2011, 29 (2), 143–148. 10.1038/nbt.1755. 21179091

[ref11] Christian M.; Cermak T.; Doyle E. L.; Schmidt C.; Zhang F.; Hummel A.; Bogdanove A. J.; Voytas D. F. Targeting DNA Double-Strand Breaks with TAL Effector Nucleases. Genetics 2010, 186 (2), 757–761. 10.1534/genetics.110.120717. 20660643 PMC2942870

[ref12] Bogdanove A. J.; Voytas D. F. TAL Effectors: Customizable Proteins for DNA Targeting. Science 2011, 333 (6051), 1843–1846. 10.1126/science.1204094. 21960622

[ref13] Cong L.; Ran F. A.; Cox D.; Lin S.; Barretto R.; Habib N.; Hsu P. D.; Wu X.; Jiang W.; Marraffini L. A.; Zhang F. Multiplex Genome Engineering Using CRISPR/Cas Systems. Science 2013, 339 (6121), 819–823. 10.1126/science.1231143. 23287718 PMC3795411

[ref14] Mali P.; Yang L.; Esvelt K. M.; Aach J.; Guell M.; DiCarlo J. E.; Norville J. E.; Church G. M. RNA-Guided Human Genome Engineering via Cas9. Science 2013, 339 (6121), 823–826. 10.1126/science.1232033. 23287722 PMC3712628

[ref15] Jinek M.; Chylinski K.; Fonfara I.; Hauer M.; Doudna J. A.; Charpentier E. A Programmable Dual-RNA-Guided DNA Endonuclease in Adaptive Bacterial Immunity. Science 2012, 337 (6096), 816–821. 10.1126/science.1225829. 22745249 PMC6286148

[ref16] Guo J.; Gaj T.; Barbas C. F. Directed Evolution of an Enhanced and Highly Efficient FokI Cleavage Domain for Zinc Finger Nucleases. J. Mol. Biol. 2010, 400 (1), 96–107. 10.1016/j.jmb.2010.04.060. 20447404 PMC2885538

[ref17] Kleinstiver B. P.; Wang L.; Wolfs J. M.; Kolaczyk T.; McDowell B.; Wang X.; Schild-Poulter C.; Bogdanove A. J.; Edgell D. R. The I-TevI Nuclease and Linker Domains Contribute to the Specificity of Monomeric TALENs. G3 (Bethesda) 2014, 4 (6), 1155–1165. 10.1534/g3.114.011445. 24739648 PMC4065259

[ref18] Tsai S. Q.; Wyvekens N.; Khayter C.; Foden J. A.; Thapar V.; Reyon D.; Goodwin M. J.; Aryee M. J.; Joung J. K. Dimeric CRISPR RNA-Guided FokI Nucleases for Highly Specific Genome Editing. Nat. Biotechnol. 2014, 32 (6), 569–576. 10.1038/nbt.2908. 24770325 PMC4090141

[ref19] Wolfs J. M.; Hamilton T. A.; Lant J. T.; Laforet M.; Zhang J.; Salemi L. M.; Gloor G. B.; Schild-Poulter C.; Edgell D. R. Biasing Genome-Editing Events toward Precise Length Deletions with an RNA-Guided TevCas9 Dual Nuclease. Proc. Natl. Acad. Sci. U. S. A. 2016, 113 (52), 14988–14993. 10.1073/pnas.1616343114. 27956611 PMC5206545

[ref20] Wolfs J. M.; DaSilva M.; Meister S. E.; Wang X.; Schild-Poulter C.; Edgell D. R. MegaTevs: Single-Chain Dual Nucleases for Efficient Gene Disruption. Nucleic Acids Res. 2014, 42 (13), 8816–8829. 10.1093/nar/gku573. 25013171 PMC4117789

[ref21] English M. A.; Soenksen L. R.; Gayet R. V.; de Puig H.; Angenent-Mari N. M.; Mao A. S.; Nguyen P. Q.; Collins J. J. Programmable CRISPR-Responsive Smart Materials. Science 2019, 365 (6455), 780–785. 10.1126/science.aaw5122. 31439791

[ref22] McVey C.; Huang F.; Elliott C.; Cao C. Endonuclease Controlled Aggregation of Gold Nanoparticles for the Ultrasensitive Detection of Pathogenic Bacterial DNA. Biosens Bioelectron 2017, 92, 502–508. 10.1016/j.bios.2016.10.072. 27825885

[ref23] Kaminski M. M.; Abudayyeh O. O.; Gootenberg J. S.; Zhang F.; Collins J. J. CRISPR-Based Diagnostics. Nat. Biomed Eng. 2021, 5 (7), 643–656. 10.1038/s41551-021-00760-7. 34272525

[ref24] Fan C.; Davison P. A.; Habgood R.; Zeng H.; Decker C. M.; Gesell Salazar M.; Lueangwattanapong K.; Townley H. E.; Yang A.; Thompson I. P.; Ye H.; Cui Z.; Schmidt F.; Hunter C. N.; Huang W. E. Chromosome-Free Bacterial Cells Are Safe and Programmable Platforms for Synthetic Biology. Proc. Natl. Acad. Sci. U. S. A. 2020, 117 (12), 6752–6761. 10.1073/pnas.1918859117. 32144140 PMC7104398

[ref25] Jain S.; Shukla S.; Yang C.; Zhang M.; Fatma Z.; Lingamaneni M.; Abesteh S.; Lane S. T.; Xiong X.; Wang Y.; Schroeder C. M.; Selvin P. R.; Zhao H. TALEN Outperforms Cas9 in Editing Heterochromatin Target Sites. Nat. Commun. 2021, 12 (1), 606 10.1038/s41467-020-20672-5. 33504770 PMC7840734

[ref26] Bhardwaj A.; Nain V. TALENs—an Indispensable Tool in the Era of CRISPR: A Mini Review. Journal of Genetic Engineering and Biotechnology 2021, 19 (1), 125 10.1186/s43141-021-00225-z. 34420096 PMC8380213

[ref27] Edgell D. R.; Gibb E. A.; Belfort M. Mobile DNA Elements in T4 and Related Phages. Virol J. 2010, 7 (1), 290 10.1186/1743-422X-7-290. 21029434 PMC2988022

[ref28] Bell-Pedersen D.; Quirk S. M.; Bryk M.; Belfort M. I-TevI, the Endonuclease Encoded by the Mobile Td Intron, Recognizes Binding and Cleavage Domains on Its DNA Target. Proc. Natl. Acad. Sci. U. S. A. 1991, 88 (17), 7719–7723. 10.1073/pnas.88.17.7719. 1881913 PMC52374

[ref29] Kleinstiver B. P.; Wolfs J. M.; Kolaczyk T.; Roberts A. K.; Hu S. X.; Edgell D. R. Monomeric Site-Specific Nucleases for Genome Editing. Proc. Natl. Acad. Sci. U. S. A. 2012, 109 (21), 8061–8066. 10.1073/pnas.1117984109. 22566637 PMC3361397

[ref30] Dunin-Horkawicz S.; Feder M.; Bujnicki J. M. Phylogenomic Analysis of the GIY-YIG Nuclease Superfamily. BMC Genomics 2006, 7 (1), 98 10.1186/1471-2164-7-98. 16646971 PMC1564403

[ref31] Kowalski J. C.; Belfort M.; Stapleton M. A.; Holpert M.; Dansereau J. T.; Pietrokovski S.; Baxter S. M.; Derbyshire V. Configuration of the Catalytic GIY-YIG Domain of Intron Endonuclease I-TevI: Coincidence of Computational and Molecular Findings. Nucleic Acids Res. 1999, 27 (10), 2115–2125. 10.1093/nar/27.10.2115. 10219084 PMC148431

[ref32] Dean A. B.; Stanger M. J.; Dansereau J. T.; Van Roey P.; Derbyshire V.; Belfort M. Zinc Finger as Distance Determinant in the Flexible Linker of Intron Endonuclease I-TevI. Proc. Natl. Acad. Sci. U. S. A. 2002, 99 (13), 8554–8561. 10.1073/pnas.082253699. 12077294 PMC124309

[ref33] Liu Q.; Dansereau J. T.; Puttamadappa S. S.; Shekhtman A.; Derbyshire V.; Belfort M. Role of the Interdomain Linker in Distance Determination for Remote Cleavage by Homing Endonuclease I-TevI. J. Mol. Biol. 2008, 379 (5), 1094–1106. 10.1016/j.jmb.2008.04.047. 18499124 PMC2699217

[ref34] Mueller J. E.; Smith D.; Bryk M.; Belfort M. Intron-Encoded Endonuclease I-TevI Binds as a Monomer to Effect Sequential Cleavage via Conformational Changes in the Td Homing Site. EMBO J. 1995, 14 (22), 5724–5735. 10.1002/j.1460-2075.1995.tb00259.x. 8521829 PMC394687

[ref35] Bryk M.; Quirk S. M.; Mueller J. E.; Loizos N.; Lawrence C.; Belfort M. The Td Intron Endonuclease I-TevI Makes Extensive Sequence-Tolerant Contacts across the Minor Groove of Its DNA Target. EMBO J. 1993, 12 (5), 2141–2149. 10.1002/j.1460-2075.1993.tb05862.x. 8491202 PMC413435

[ref36] Edgell D. R.; Derbyshire V.; van Roey P.; LaBonne S.; Stanger M. J.; Li Z.; Boyd T. M.; Shub D. A.; Belfort M. Intron-Encoded Homing Endonuclease I-TevI Also Functions as a Transcriptional Autorepressor. Nat. Struct Mol. Biol. 2004, 11 (10), 936–944. 10.1038/nsmb823. 15361856

[ref37] van Roey P.; Meehan L.; Kowalski J. C.; Belfort M.; Derbyshire V. Catalytic Domain Structure and Hypothesis for Function of GIY-YIG Intron Endonuclease I-TevI. Nat. Struct. Biol. 2002, 10.1038/nsb853. 12379841

[ref38] Pereira C. V.; Bacman S. R.; Arguello T.; Zekonyte U.; Williams S. L.; Edgell D. R.; Moraes C. T. MitoTev-TALE: A Monomeric DNA Editing Enzyme to Reduce Mutant Mitochondrial DNA Levels. EMBO Mol. Med. 2018, 10 (9), e8084 10.15252/emmm.201708084. 30012581 PMC6127889

[ref39] McCue A. C.; Kuhlman B. Design and Engineering of Light-Sensitive Protein Switches. Curr. Opin Struct Biol. 2022, 74, 102377 10.1016/j.sbi.2022.102377. 35461160 PMC9968517

[ref40] Zayner J. P.; Antoniou C.; Sosnick T. R. The Amino-Terminal Helix Modulates Light-Activated Conformational Changes in AsLOV2. J. Mol. Biol. 2012, 419 (1–2), 61–74. 10.1016/j.jmb.2012.02.037. 22406525 PMC3338903

[ref41] Herrou J.; Crosson S. Function, Structure and Mechanism of Bacterial Photosensory LOV Proteins. Nat. Rev. Microbiol 2011, 9 (10), 713–723. 10.1038/nrmicro2622. 21822294 PMC3286519

[ref42] Losi A.; Gärtner W. Solving Blue Light Riddles: New Lessons from Flavin-Binding LOV Photoreceptors. Photochem. Photobiol. 2017, 93 (1), 141–158. 10.1111/php.12674. 27861974

[ref43] Yao X.; Rosen M. K.; Gardner K. H. Estimation of the Available Free Energy in a LOV2-Jα Photoswitch. Nat. Chem. Biol. 2008, 4 (8), 491–497. 10.1038/nchembio.99. 18604202 PMC2597337

[ref44] Strickland D.; Moffat K.; Sosnick T. R. Light-Activated DNA Binding in a Designed Allosteric Protein. Proc. Natl. Acad. Sci. U. S. A. 2008, 105 (31), 10709–10714. 10.1073/pnas.0709610105. 18667691 PMC2504796

[ref45] Strickland D.; Yao X.; Gawlak G.; Rosen M. K.; Gardner K. H.; Sosnick T. R. Rationally Improving LOV Domain-Based Photoswitches. Nat. Methods 2010, 7 (8), 623–626. 10.1038/nmeth.1473. 20562867 PMC2914111

[ref46] Kalvaitis M. E.; Johnson L. A.; Mart R. J.; Rizkallah P.; Allemann R. K. A Noncanonical Chromophore Reveals Structural Rearrangements of the Light-Oxygen-Voltage Domain upon Photoactivation. Biochemistry 2019, 58 (22), 2608–2616. 10.1021/acs.biochem.9b00255. 31082213 PMC7007005

[ref47] Wu W. Intein-Mediated Purification of Cytotoxic Endonuclease I-TevI by Insertional Inactivation and PH-Controllable Splicing. Nucleic Acids Res. 2002, 30 (22), 4864–4871. 10.1093/nar/gkf621. 12433989 PMC137169

[ref48] Doyon J. B.; Pattanayak V.; Meyer C. B.; Liu D. R. Directed Evolution and Substrate Specificity Profile of Homing Endonuclease I-SceI. J. Am. Chem. Soc. 2006, 128 (7), 2477–2484. 10.1021/ja057519l. 16478204

[ref49] Chen Z.; Zhao H. A Highly Sensitive Selection Method for Directed Evolution of Homing Endonucleases. Nucleic Acids Res. 2005, 33 (18), e154 10.1093/nar/gni148. 16214805 PMC1253837

[ref50] Brocken D. J. W.; Tark-Dame M.; Dame R. T. DCas9: A Versatile Tool for Epigenome Editing. Curr. Issues Mol. Biol. 2018, 15–32. 10.21775/cimb.026.015. 28879853

[ref51] Beasock D.; Ha A.; Halman J.; Panigaj M.; Wang J.; Dokholyan N. V.; Afonin K. A. Break to Build: Isothermal Assembly of Nucleic Acid Nanoparticles (NANPs) *via* Enzymatic Degradation. Bioconjug Chem. 2023, 34 (6), 1139–1146. 10.1021/acs.bioconjchem.3c00167. 37293781 PMC10288440

[ref52] Aye S.; Sato Y. Therapeutic Applications of Programmable DNA Nanostructures. Micromachines (Basel) 2022, 13 (2), 315 10.3390/mi13020315. 35208439 PMC8876680

[ref53] Engler C.; Kandzia R.; Marillonnet S. A One Pot, One Step, Precision Cloning Method with High Throughput Capability. PLoS One 2008, 3 (11), e3647 10.1371/journal.pone.0003647. 18985154 PMC2574415

