# Supplementary material for: A Photoresponsive Homing Endonuclease for Programmed DNA Cleavage
Source: ACS Synth Biol. 2023 Dec 7;13(1):195–205. doi: 10.1021/acssynbio.3c00425 (PMC10804406; doi:10.1021/acssynbio.3c00425)
Supplement: Supplementary file 1 — sb3c00425_si_001.pdf [file sb3c00425_si_001.pdf]

Supporting Information

A Photo-responsive Homing Endonuclease for Programmed DNA Cleavage

Luke A. Johnson, Robert J. Mart[1] and Rudolf K. Allemann*
School of Chemistry, Cardiff University, Main Building, Park Place, CF10 3AT, U.K.
[1] Current address, Ochre Bio Ltd, Hayakawa Building Oxford Science Park, Edmund Halley Road, Oxford, England, OX4 4GB, U.K.
* Corresponding author allemannRK@cardiff.ac.uk

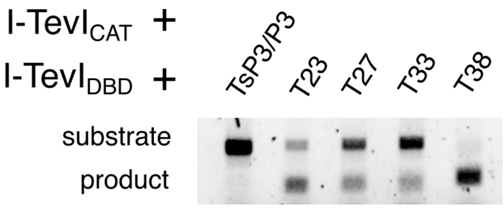

Figure S1: 1.5 - 2% agarose gel showing single cleavage points comparing the activity of I-TevI$_{CAT}$(1-170)-Au1a and Au1a-I-TevI$_{DBD}$ parts in a 1:1 ratio against different substrates where the I-TevI homing site was reduced in length by 15 (T23), 11 (T27) and 5 (T33) bp from the 3' end. Each 2-minute reaction was performed using 13 nM cy5.5 labelled substrate and 125 nM enzyme.

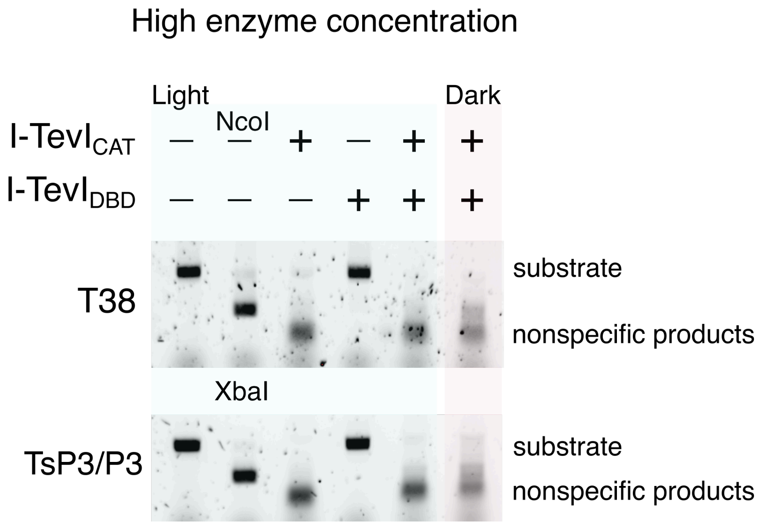

Figure S2: 1.5 - 2% agarose gel showing the effect of high enzyme concentration (1.25 μM) on the specificity of the split I-TevI Au1a system. Different combinations of I-TevI$_{CAT}$(1-170)-Au1a and Au1a-I-TevI$_{DBD}$ parts at 1.25 μM were compared under blue light and dark conditions for 13 nM T38 substrate containing the I-TevI homing site and the TsP3/P3 control. Under these conditions of 1.25 μM I-TevI$_{CAT}$(1-170)-Au1a small nonspecific products under both dark and illuminated conditions for both substrates were observed.

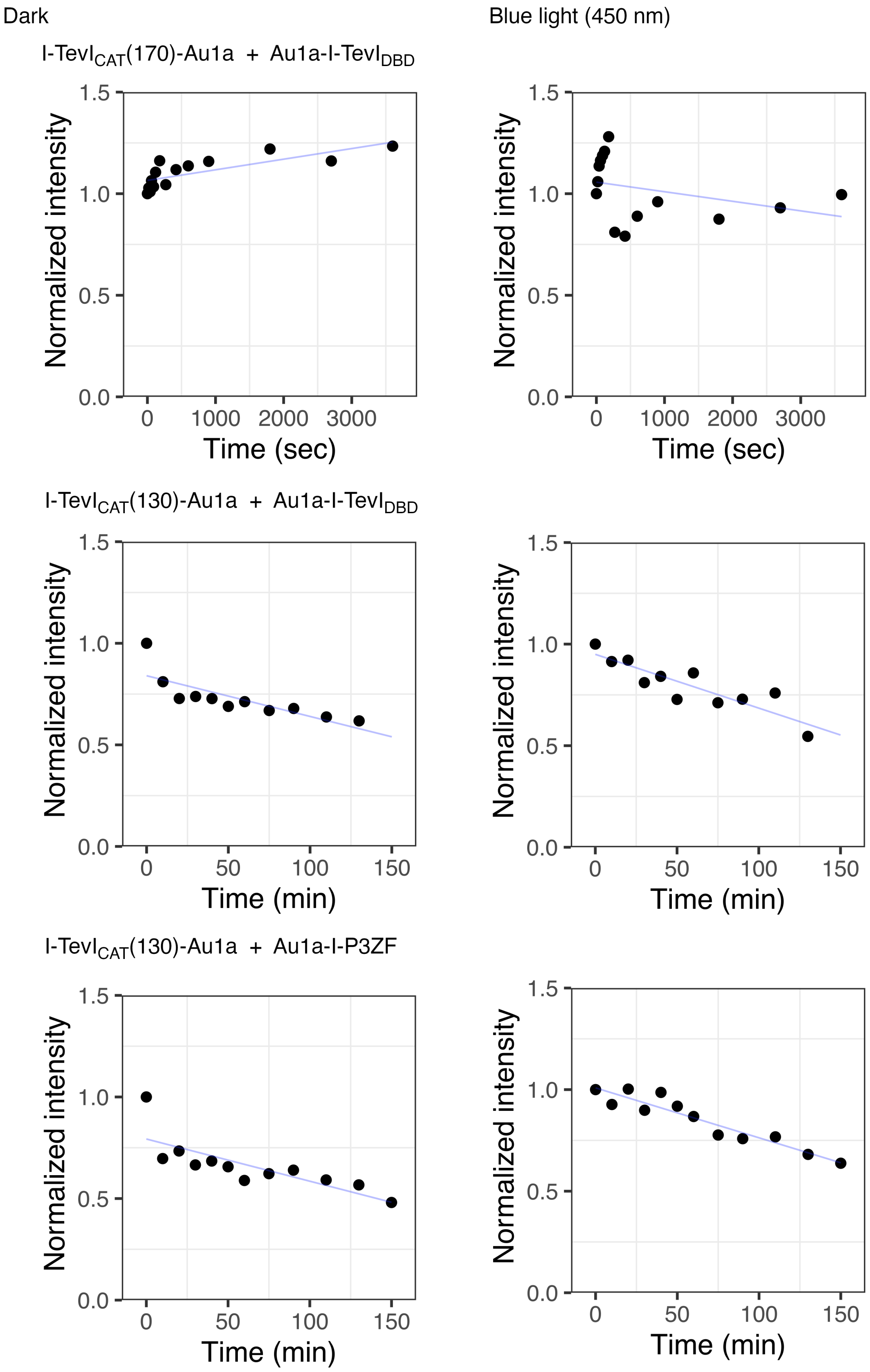

Figure S3: Overall intensity changes for the total volume of substrate and product bands for each single-turnover cleavage assay in the main text for each set of constructs under dark and blue light conditions. Each plot is normalised relative to the volume of the first lane. Gel loading errors are not accounted for. First four plots are with 13 nM T38 substrate, bottom two plots are with 13 nM TsP3/P3. For I-TevI$_{CAT}$(130)-Au1a there is a linear loss of fluorescence intensity across the timecourse under both illuminated and dark state conditions indicating non-specific cleavage occurs. Loss of cleavage fidelity is not observed for I-TevI$_{CAT}$(170)-Au1a.

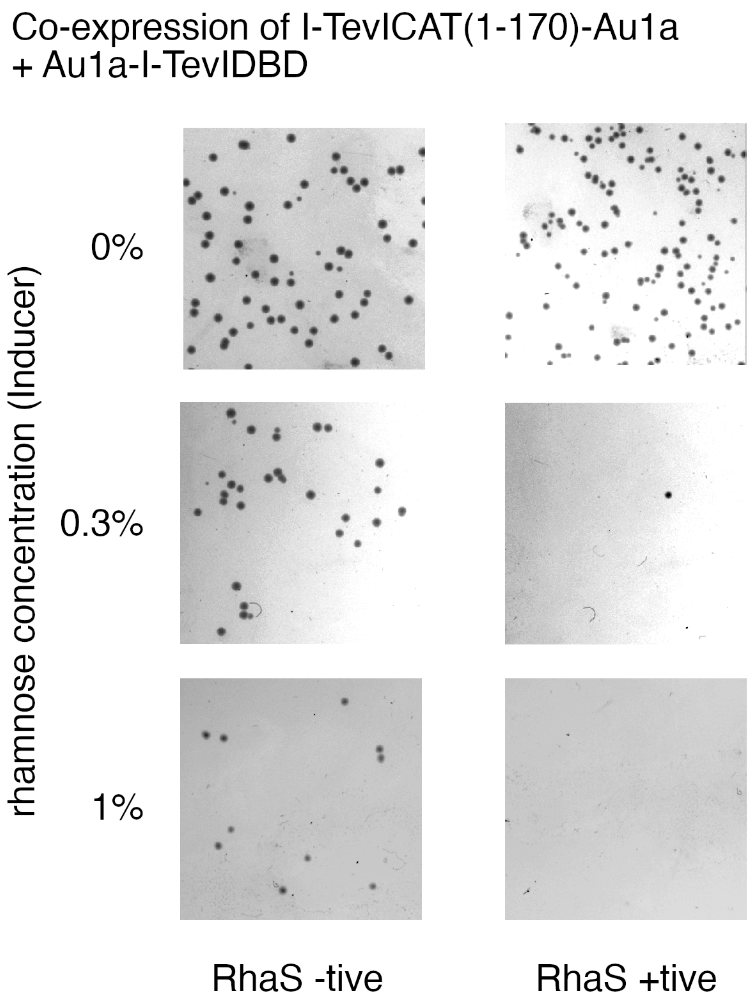

20 A

Figure S4: LB-agar plates showing representative number of *E. coli* colonies after transformation with plasmids containing the genes for I-TevI(1-170)-Au1a and Au1a-I-TevIDBD under the control of the rhamnose promoter. Plasmid either lacked (left) or contained (right) the additional gene encoding the RhaS activator of the rhamnose promoter under J23107 Anderson promoter which increases expression level. Expression was induced with rhamnose for 1 hour under blue light. The transformation efficiency reduced for both plasmids with increasing levels of rhamnose. The effect was greater for RhaS positive plasmid where expression level is expected to be higher.

Gene and protein sequences of designed endonuclease constructs

I-TevI$_{CAT}$(1-170)-Au1a

atgggcagcagccatcatcatcatcatcacagcagcggcctggtgccgcgcggcagccatatggaaaacctgtattttcagtccatgaaaagcggaatttatcagattaaaaatactttaaacaa
taaagtatatgtaggaagtgctaaagattttgaaaagagatggaagaggcattttaaagatttagaaaaaggatgccattcttctataaaacttcagaggtcttttaacaaacatggtaatgtgtttga
atgttctattttggaagaaattccatatgagaaagatttgattattgaacgagaaaatttttggattaaagagcttaattctaaaattaatggatacaatattgctgatgcaacgtttggtgatacatgttcta
cgcatccattaaaagaagaaattattaagaaacgttctgaaactgttaaagctaagatgcttaaacttggacctgatggtcggaaagctctttacagtaaacccggaagtaaaaacgggcgttgg
aatccagaaacccataagttttgtaagtgcggtgttcgcatacaaacttctgcttatacttgtagtaaatgcagaaatcgttccggaaacactgcgactcgcactctggatgatccggattacagcct
ggtgaaggccctgcaaaccgctcagcagaacttcgttatctctgatccgagcattccggacaacccgatcgtatatgctagccaaggcttcctgaccctgactggttacgcgctgagcgaagtact
gggtcgtaactgtcgcttcctgcaaggtccagaaaccgatccgaaagctgtagagaaagttcgtaaaggtctggaacgtggcgaggatactaccgttgtactgctgaactatcgcaaagacggtt
ctaccttctggaaccaactgtttatcgcagctctgcgtgacggcgaaggtaacgtagtgaactacctgggcgttcagtgcaaagtatccgaggactatgctaaagcgttcctgaagaatgaggag
aacgagaagtga

MGSSHHHHHHSSGLVPRGSHMENLYFQSMKSGIYQIKNTLNNKVYVGSAKDFEKRWKRHFKDLEKGCHSSIKLQRSFNKHGNVFECSIL
EEIPYEKDLIIERENFWIKELNSKINGYNIADATFGDTCSTHPLKEEIIKKRSETVKAKMLKLGPDGRKALYSKPGSKNGRWNPETHKFCKC
GVRIQTSAYTCSKCRNRSGNTATRTLDDPDYSLVKALQTAQQNFVISDPSIPDNPIVYASQGFLTLTGYALSEVLGRNCRFLQGPETDPKA
VEKVRKGLERGEDTTVVLLNYRKDGSTFWNQLFIAALRDGEGNVVNYLGVQCKVSEDYAKAFLKNEENEK*

I-TevI$_{CAT}$(1-130)-Au1a

atgggcagcagccatcatcatcatcatcacagcagcggcctggtgccgcgcggcagccatatggaaaacctgtattttcagtccatgaaaagcggaatttatcagattaaaaatactttaaacaataa
agtatatgtaggaagtgctaaagattttgaaaagagatggaagaggcattttaaagatttagaaaaaggatgccattcttctataaaacttcagaggtcttttaacaaacatggtaatgtgtttgaatg
ttctattttggaagaaattccatatgagaaagatttgattattgaacgagaaaatttttggattaaagagcttaattctaaaattaatggatacaatattgctgatgcaacgtttggtgatacatgttctacg
catccattaaaagaagaaattattaagaaacgttctgaaactgttaaagctaagatgcttaaacttggacctgatggtcggaaagctggaaacactgcgactcgcactctggatgatccggattac
agcctggtgaaggccctgcaaaccgctcagcagaacttcgttatctctgatccgagcattccggacaacccgatcgtatatgctagccaaggcttcctgaccctgactggttacgcgctgagcga
agtactgggtcgtaactgtcgcttcctgcaaggtccagaaaccgatccgaaagctgtagagaaagttcgtaaaggtctggaacgtggcgaggatactaccgttgtactgctgaactatcgcaaag
acggttctaccttctggaaccaactgtttatcgcagctctgcgtgacggcgaaggtaacgtagtgaactacctgggcgttcagtgcaaagtatccgaggactatgctaaagcgttcctgaagaatg
aggagaacgagaagtga

MGSSHHHHHHSSGLVPRGSHMENLYFQSMKSGIYQIKNTLNNKVYVGSAKDFEKRWKRHFKDLEKGCHSSIKLQRSFNKHGNVFECSILE
EIPYEKDLIIERENFWIKELNSKINGYNIADATFGDTCSTHPLKEEIIKKRSETVKAKMLKLGPDGRKAGNTATRTLDDPDYSLVKALQTAQQ
NFVISDPSIPDNPIVYASQGFLTLTGYALSEVLGRNCRFLQGPETDPKAVEKVRKGLERGEDTTVVLLNYRKDGSTFWNQLFIAALRDGEG
NVVNYLGVQCKVSEDYAKAFLKNEENEK*

Au1a-I-TevI$_{DBD}$

atgggcagcagccatcatcatcatcatcacagcagcggcctggtgccgcgcggcagccatatggaaaacctgtattttcagtccgattacagcctggtgaaggccctgcaaaccgctcagcag
aacttcgttatctctgatccgagcattccggacaacccgatcgtatatgctagccaaggcttcctgaccctgactggttacgcgctgagcgaagtactgggtcgtaactgtcgcttcctgcaaggtcc
agaaaccgatccgaaagctgtagagaaagttcgtaaaggtctggaacgtggcgaggatactaccgttgtactgctgaactatcgcaaagacggttctaccttctggaaccaactgtttatcgcag
ctctgcgtgacggcgaaggtaacgtagtgaactacctgggcgttcagtgcaaagtatccgaggactatgctaaagcgttcctgaagaatgaggagaacgagaacggatcaggtgaaaataatt
cattctttaatcataagcattcagacataactaaatctaaaatatcagaaaagatgaaaggtaaaaagcctagtaatattaaaaagatttcatgtgatggggttatttttgattgtgcagcagatgcag
ctagacattttaaaatttcgtctggattagttacttatcgtgtaaaatctgataaatggaattggttctacataaatgcctga

MGSSHHHHHHSSGLVPRGSHMENLYFQSDYSLVKALQTAQQNFVISDPSIPDNPIVYASQGFLTLTGYALSEVLGRNCRFLQGPETDPKA
VEKVRKGLERGEDTTVVLLNYRKDGSTFWNQLFIAALRDGEGNVVNYLGVQCKVSEDYAKAFLKNEENENGSGENNSFFNHKHSDITKS
KISEKMKGKKPSNIKKISCDGVIFDCAADAARHFKISSGLVTYRVKSDKWNWFYINA*

Au1a-P3ZF

atgggcagcagccatcatcatcatcatcacagcagcggcctggtgccgcgcggcagccatatggaaaacctgtattttcagtccgattacagcctggtgaaggccctgcaaaccgctcagcag
aacttcgttatctctgatccgagcattccggacaacccgatcgtatatgctagccaaggcttcctgaccctgactggttacgcgctgagcgaagtactgggtcgtaactgtcgcttcctgcaaggtcc
agaaaccgatccgaaagctgtagagaaagttcgtaaaggtctggaacgtggcgaggatactaccgttgtactgctgaactatcgcaaagacggttctaccttctggaaccaactgtttatcgcag
ctctgcgtgacggcgaaggtaacgtagtgaactacctgggcgttcagtgcaaagtatccgaggactatgctaaagcgttcctgaagaatgaggagaacgagaacggagagaagccctatgct
tgtccggaatgtggtaagtccttcagccgcagcgatgacctggtgcgccaccagcgtacccacacgggtgaaaaaccgtataaatgcccagagtgcggcaaatcttttagtcgcagcgatgtgc
tggtgcgccatcaacgcactcatactggcgagaagccatacaaatgtccagaatgtggcaagtctttcagccagagcggcgatctgcgccgccaccaacgtactcacaccggtaaaaaaactt
ga

MGSSHHHHHHSSGLVPRGSHMENLYFQSDYSLVKALQTAQQNFVISDPSIPDNPIVYASQGFLTLTGYALSEVLGRNCRFLQGPETDPKA
VEKVRKGLERGEDTTVVLLNYRKDGSTFWNQLFIAALRDGEGNVVNYLGVQCKVSEDYAKAFLKNEENENGEKPYACPECGKSFSRSD
DLVRHQRTHTGEKPYKCPECGKSFSRSDVLVRHQRTHTGEKPYKCPECGKSFSQSGDLRRHQRTHTGKKT*

Blue: His_6 tag and TEV protease site. Red: LOV domain. Green: I-TevI nuclease catalytic domain. Yellow: I-TevI nuclease DNA binding domain (residues 171-245).

Substrate DNA sequences

T38
Cy5.5 - 5’
tggccagtgcacgtctgctgtcagataaagtctcccgtgaactttacccggtggtgcatatcggggatgaaagctggcgcatgatgaccac
cgatatggccagtgtgccggtctccgttatcggggaagaagtggctgatctcagccaccgcgaaaatgacatcaaaaacgccattaacct
gatgttttggggaatataatctagtagaccatggtcttaaacggtagacccaagaaaacatctactgagcgttgactagacgccactgcac
gcgtgcagtggcgctagggataacagggtaatataggcatgcaagcttggctgttttggcggatgagagaagattttcagcctgatacaga
ttaaatcagaacgcagaagcggtctgataaaacagaatttgcctggcggcagtagcgcggtggtcccacctgaccccatgccgaactca
gaagtgaaacgccgtagcgccgatggtagtgtggggtctccccatgcgagagtagggaactgccaggcatcaaataaaacgaaaggc
tcagtcgaaagactgggcctttcgttttatctgttgtttgtcggtgaacgctctcctgagtaggacaaatccgccgggagcgg 3’

T38ΔP3
Cy5.5 - 5’
tggccagtgcacgtctgctgtcagataaagtctcccgtgaactttacccggtggtgcatatcggggatgaaagctggcgcatgatgaccac
cgatatggccagtgtgccggtctccgttatcggggaagaagtggctgatctcagccaccgcgaaaatgacatcaaaaacgccattaacct
gatgttttggggaatataatctagtagaccatggtcttaaacggtagacccaagaaaacatctactgagcgttgactagactagggataac
agggtaatataggcatgcaagcttggctgttttggcggatgagagaagattttcagcctgatacagattaaatcagaacgcagaagcggtc
tgataaaacagaatttgcctggcggcagtagcgcggtggtcccacctgaccccatgccgaactcagaagtgaaacgccgtagcgccga
tggtagtgtggggtctccccatgcgagagtagggaactgccaggcatcaaataaaacgaaaggctcagtcgaaagactgggcctttcgtt
ttatctgttgtttgtcggtgaacgctctcctgagtaggacaaatccgccgggagcgg 3’

TsP3/P3
Cy5.5 - 5’
tggccagtgcacgtctgctgtcagataaagtctcccgtgaactttacccggtggtgcatatcggggatgaaagctggcgcatgatgaccac
cgatatggccagtgtgccggtctccgttatcggggaagaagtggctgatctcagccaccgcgaaaatgacatcaaaaacgccattaacct
gatgttttggggaatataatctagacgccactgcacgcgtgcagtggcgctagggataacagggtaatataggcatgcaagcttggctgttt
tggcggatgagagaagattttcagcctgatacagattaaatcagaacgcagaagcggtctgataaaacagaatttgcctggcggcagta
gcgcggtggtcccacctgaccccatgccgaactcagaagtgaaacgccgtagcgccgatggtagtgtggggtctccccatgcgagagta
gggaactgccaggcatcaaataaaacgaaaggctcagtcgaaagactgggcctttcgttttatctgttgtttgtcggtgaacgctctcctgag
taggacaaatccgccgggagcgg 3’

TΔP3
Cy5.5 - 5’
tggccagtgcacgtctgctgtcagataaagtctcccgtgaactttacccggtggtgcatatcggggatgaaagctggcgcatgatgaccac
cgatatggccagtgtgccggtctccgttatcggggaagaagtggctgatctcagccaccgcgaaaatgacatcaaaaacgccattaacct
gatgttttggggaatataatctagttataggtagcccatggtccatcctgattcgttagcttggctgttttggcggatgagagaagattttcagcct
gatacagattaaatcagaacgcagaagcggtctgataaaacagaatttgcctggcggcagtagcgcggtggtcccacctgaccccatgc
cgaactcagaagtgaaacgccgtagcgccgatggtagtgtggggtctccccatgcgagagtagggaactgccaggcatcaaataaaa
cgaaaggctcagtcgaaagactgggcctttcgttttatctgttgtttgtcggtgaacgctctcctgagtaggacaaatccgccgggagcgg 3’

Green: I-TevI homing site (antisense). Red: sP3/P3 zinc finger binding site (sense/antisense).

Plasmid maps for co-expression of I-TevI$_{CAT}$(1-170)-Au1a and Au1a-I-TevI$_{DBD}$

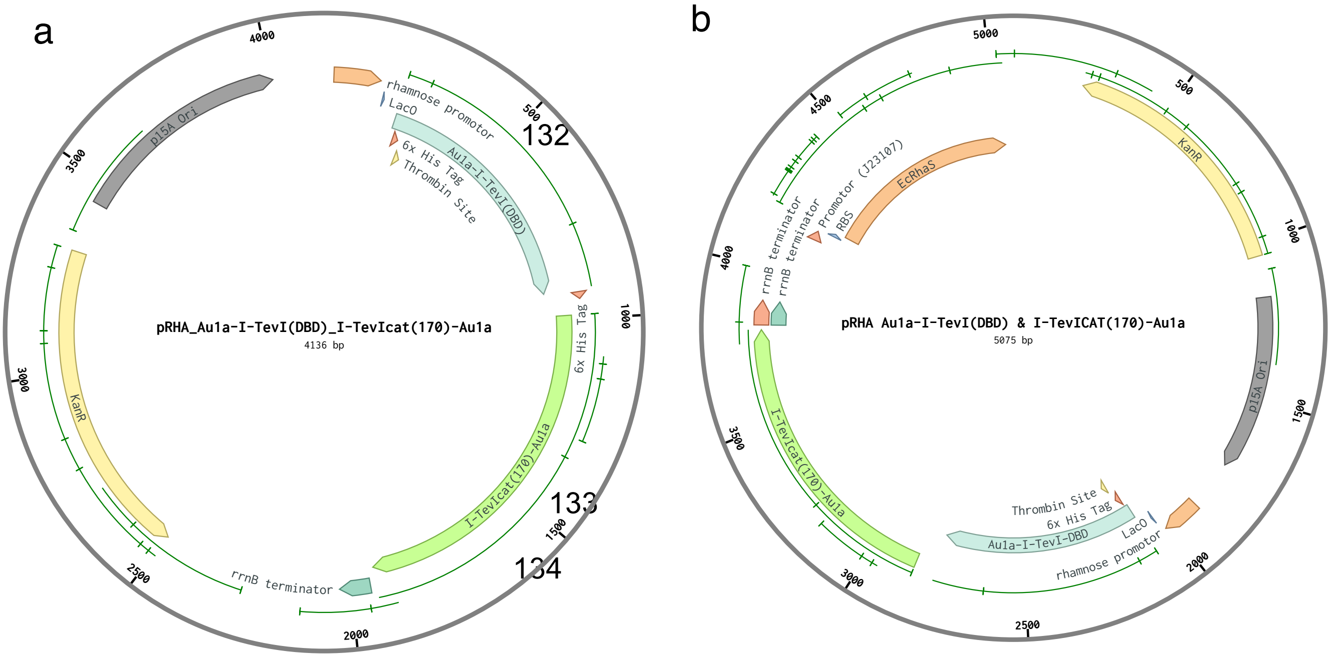

Plasmids for co-expression contain I-TevI$_{CAT}$(1-170)-Au1a and Au1a-I-TevI$_{DBD}$ parts in a single operon under the control of the rhamnose promoter Plasmid b has additional EcRhaS gene under the J23107 constitutive promoter to increase expression from the rhamnose promoter.

The operon sequence is provided in full below.

ccacaattcagcaaattgtgaacatcatcacgttcatctttccctggttgccaatggcccattttcctgtcagtaacgagaaggtcgcgtattca
ggcgctttttagactggtcgtaatgaaccatgcgagagtagcaattcccctctagaaataattttgtttaactttaagaaggagatataccatg
ggcagcagccatcatcatcatcatcacagcagcggcctggtgccgcgcggcagccatatggaaaacctgtattttcagtccgattacagc
ctggtgaaggccctgcaaaccgctcagcagaacttcgttatctctgatccgagcattccggacaacccgatcgtatatgctagccaaggctt
cctgaccctgactggttacgcgctgagcgaagtactgggtcgtaactgtcgcttcctgcaaggtccagaaaccgatccgaaagctgtaga
gaaagttcgtaaaggtctggaacgtggcgaggatactaccgttgtactgctgaactatcgcaaagacggttctaccttctggaaccaactgt
ttatcgcagctctgcgtgacggcgaaggtaacgtagtgaactacctgggcgttcagtgcaaagtatccgaggactatgctaaagcgttcct
gaagaatgaggagaacgagaacggatcaggtgaaaataattcattctttaatcataagcattcagacataactaaatctaaaatatcaga
aaagatgaaaggtaaaaagcctagtaatattaaaaagatttcatgtgatggggttatttttgattgtgcagcagatgcagctagacattttaaa
atttcgtctggattagttacttatcgtgtaaaatctgataaatggaattggttctacataaatgcctgatactcgagcaccaccaccaccaccac
tgagatccggctgctaacaaagcccgaaaggaggctgagttatgaaaagcggaatttatcagattaaaaatactttaaacaataaagtat
atgtaggaagtgctaaagattttgaaaagagatggaagaggcattttaaagatttagaaaaaggatgccattcttctataaaacttcagagg
tcttttaacaaacatggtaatgtgtttgaatgttctattttggaagaaattccatatgagaaagatttgattattgaacgagaaaatttttggattaa
agagcttaattctaaaattaatggatacaatattgctgatgcaacgtttggtgatacatgttctacgcatccattaaaagaagaaattattaaga
aacgttctgaaactgttaaagctaagatgcttaaacttggacctgatggtcggaaagctctttacagtaaacccggaagtaaaaacgggcg
ttggaatccagaaacccataagttttgtaagtgcggtgttcgcatacaaacttctgcttatacttgtagtaaatgcagaaatcgttccggaaac
actgcgactcgcactctggatgatccggattacagcctggtgaaggccctgcaaaccgctcagcagaacttcgttatctctgatccgagcat
tccggacaacccgatcgtatatgctagccaaggcttcctgaccctgactggttacgcgctgagcgaagtactgggtcgtaactgtcgcttcct
gcaaggtccagaaaccgatccgaaagctgtagagaaagttcgtaaaggtctggaacgtggcgaggatactaccgttgtactgctgaact
atcgcaaagacggttctaccttctggaaccaactgtttatcgcagctctgcgtgacggcgaaggtaacgtagtgaactacctgggcgttcag
tgcaaagtatccgaggactatgctaaagcgttcctgaagaatgaggagaacgagaagtgatactcgagggaactgccaggcatcaaat
aaaacgaaaggctcagtcgaaagactgggcctttcgttttatctgttgtttgtcggtgaacgctctc

Red: Rhamnose promoter Green: Au1a-I-TevI$_{DBD}$ gene Orange: I-TevI$_{CAT}$(1-170)-Au1a gene

Table S1

Oligonucleotide	Sequence (5' – 3')	Template	Explanation
I-TevI 1-170cat_fwd	TCAGGTCTCGCATGAAAAGCGGAATTTATCAGATTAAAAATACTTTAAAC	PCCI I-TevI	Oligonucleotide for assembly of I-TevICAT(1-170)-Au1aLOV
I-TevI 1-170cat_rev	CATGGTCTCATCCGGAACGATTTCTGCATTTACTACAAGTATAAGCAG	PCCI I-TevI	Oligonucleotide for assembly of I-TevICAT(1-170)-Au1aLOV
I-TevI 171-245 fwd	CATGGTCTCTCGGACGTTCAGGTGAAAATAATTCATTCTTTAATCATAAGCATTCAGAC	PCCI I-TevI	Oligonucleotide for assembly of Au1aLOV-I-TevIDBD(171-245)
I-TevI 171-245 rev	CATGGTCTCAATCAGGCATTTATGTAGAACCAATTCCATTTATCAG	PCCI I-TevI	Oligonucleotide for assembly of Au1aLOV-I-TevIDBD(171-245)
Au1a LOV178-312 fwd	CATGGTCTCAAGTCCGATTACAGCCTGGTGAAGGCCCTG	*Od*Au1a	Oligonucleotide for assembly of I-TevICAT(1-170)-Au1aLOV / Au1aLOV-I-TevIDBD(171-245) / Au1aLOV-P3ZF / I-TevICAT(1-130)-Au1aLOV
Au1a LOV178-312 rev	CATGGTCTCATCCGTTCTCGTTCTCCTCATTCTTCAGGAACG	*Od*Au1a	Oligonucleotide for assembly of I-TevICAT(1-170)-Au1aLOV / Au1aLOV-I-TevIDBD(171-245) / Au1aLOV-P3ZF / I-TevICAT(1-130)-Au1aLOV
p3ZF_fwd	CATGGTCTCACGGAGAGAAGCCCTATGCTTGTCCGGAATG	pPDAZ.P3-Sharkey	Oligonucleotide for assembly of Au1aLOV-P3ZF
p3ZF_rev	CATGGTCTCAATCAAGTTTTTTTACCGGTGTGAGTACGTTGGTGG	pPDAZ.P3-Sharkey	Oligonucleotide for assembly of Au1aLOV-P3ZF
cat130_Trunc_rev	AGCTTTCCGACCATCAGGTCCAAG	I-TevI CAT(1-170)-Au1aLOV	Oligonucleotide for assembly of I-TevICAT(1-130)-Au1aLOV
cat130_Trunc_fwd	GGAAACACTGCGACTCGCACTCTG	I-TevI CAT(1-170)-Au1aLOV	Oligonucleotide for assembly of I-TevICAT(1-130)-Au1aLOV
T38 fwd	CAGACTAGTCAACGCTCAGTAGATGTTTTCTTGGGTCTACCGTTTAAGACCATGGTCTACTAGTGCA	N/A	Homing endonucleotide oligonucleotide for insertion in SpeI site.
T38 rev	TGCACTAGTAGACCATGGTCTTAAACGGTAGACCCAAGAAAACATCTACTGAGCGTTGACTAGTCTG	N/A	Homing endonucleotide oligonucleotide for insertion in SpeI site.
T33 fwd	CAGACTAGTCAACGCTCAGTAGATGTTTTCTTGGGTCTACCGGACCATGGTCTACTAGTGCA	N/A	33 bp Homing endonucleotide oligonucleotide for insertion in SpeI site.
T33 rev	TGCACTAGTAGACCATGGTCCGGTAGACCCAAGAAAACATCTACTGAGCGTTGACTAGTCTG	N/A	33 bp Homing endonucleotide oligonucleotide for insertion in SpeI site.
T27 fwd	CAGACTAGTCAACGCTCAGTAGATGTTTTCTTGGGTGACCATGGTCTACTAGTGCA	N/A	27 bp Homing endonucleotide oligonucleotide for insertion in SpeI site.
T27 rev	TGCACTAGTAGACCATGGTCACCCAAGAAAACATCTACTGAGCGTTGACTAGTCTG	N/A	27 bp Homing endonucleotide oligonucleotide for insertion in SpeI site.
T23 fwd	CAGACTAGTCAACGCTCAGTAGATGTTTTCTTGACCATGGTCTACTAGTGCA	N/A	23 bp Homing endonucleotide oligonucleotide for insertion in SpeI site.
T23 rev	TGCACTAGTAGACCATGGTCAAGAAAACATCTACTGAGCGTTGACTAGTCTG	N/A	23 bp Homing endonucleotide oligonucleotide for insertion in SpeI site.

TΔP3 fwd	CTAGTTATAGGTAGCCCATGGTCCATC CTGATTCGTT	N/A	Restriction enzyme ligation oligonucleotide for removal of P3 site(XbaI/HindIII)
TΔP3 rev	ATTATCCATCGGGTACCAGGTAGGACT AAGCAATCGA	N/A	Restriction enzyme ligation oligonucleotide for removal of P3 site (XbaI/HindIII)
T38_noP3_fwd	CTAGGGATAACAGGGTAATATAGGCAT GCAAGC	T38	Blunt-end ligation oligonucleotide for removal of P3 site from T38
T38_noP3_rev	TCTAGTCAACGCTCAGTAGATGTTTTCT TGGG	T38	Blunt-end ligation oligonucleotide for removal of P3 site from T38
Sequencing-fwd nuclease target	TGATATTATTGACACGCCCG	N/A	Sequencing oligonucleotide
Fluoro_Target_fwd	Cyanine 5.5 - TGGCCAGTGCACGTCTGCTGTCAG	T38, TsP3/P3, TΔP3, T38ΔP3	Fluorescent oligonucleotide for synthesising 600 bp substrate.
Fluoro_Target_rev	CCGCTCCCGGCGGATTTGTCCTAC	T38 TsP3/P3, TΔP3 T38ΔP3	Oligonucleotide for synthesising 600 bp substrate.

169
